# Temperature desynchronizes sugar and organic acid metabolism in ripening grapevine fruits and remodels their transcriptome

**DOI:** 10.1186/s12870-016-0850-0

**Published:** 2016-07-20

**Authors:** Markus Rienth, Laurent Torregrosa, Gautier Sarah, Morgane Ardisson, Jean-Marc Brillouet, Charles Romieu

**Affiliations:** Montpellier SupAgro-INRA, UMR AGAP-DAAV Amélioration Génétique et Adaptation des Plantes méditerranéennes et tropicales-Diversité, Adaptation et Amélioration de la Vigne, 2 place Pierre Viala, Montpellier, 34060 France; Fondation Jean Poupelain, 30 Rue Gâte Chien, Javrezac, 16100 France; CHANGINS, haute école de viticulture et œnologie, 50 route de Duillier, 1260 Nyon, Switzerland; INRA Montpellier UMR SPO- Science pour l’œnologie, 2 place, Pierre Viala, Montpellier, 34060 France

**Keywords:** Grapevine, Fruit development, Temperature stress, Malic acid, Secondary metabolism, RNA-Seq, Microvine

## Abstract

**Background:**

Fruit composition at harvest is strongly dependent on the temperature during the grapevine developmental cycle. This raises serious concerns regarding the sustainability of viticulture and the socio-economic repercussions of global warming for many regions where the most heat-tolerant varieties are already cultivated. Despite recent progress, the direct and indirect effects of temperature on fruit development are far from being understood. Experimental limitations such as fluctuating environmental conditions, intra-cluster heterogeneity and the annual reproductive cycle introduce unquantifiable biases for gene expression and physiological studies with grapevine. In the present study, DRCF grapevine mutants (microvine) were grown under several temperature regimes in duly-controlled environmental conditions. A singly berry selection increased the accuracy of fruit phenotyping and subsequent gene expression analyses. The physiological and transcriptomic responses of five key stages sampled simultaneously at day and nighttime were studied by RNA-seq analysis.

**Results:**

A total of 674 millions reads were sequenced from all experiments. Analysis of differential expression yielded in a total of 10 788 transcripts modulated by temperature. An acceleration of green berry development under higher temperature was correlated with the induction of several candidate genes linked to cell expansion. High temperatures impaired tannin synthesis and degree of galloylation at the transcriptomic levels. The timing of malate breakdown was delayed to mid-ripening in transgressively cool conditions, revealing unsuspected plasticity of berry primary metabolism. Specific ATPases and malate transporters displayed development and temperature-dependent expression patterns, besides less marked but significant regulation of other genes in the malate pathway.

**Conclusion:**

The present study represents, to our knowledge the first abiotic stress study performed on a fleshy fruits model using RNA-seq for transcriptomic analysis. It confirms that a careful stage selection and a rigorous control of environmental conditions are needed to address the long-term plasticity of berry development with respect to temperature. Original results revealed temperature-dependent regulation of key metabolic processes in the elaboration of berry composition. Malate breakdown no longer appears as an integral part of the veraison program, but as possibly triggered by an imbalance in cytoplasmic sugar, when efficient vacuolar storage is set on with ripening, in usual temperature conditions. Furthermore, variations in heat shock responsive genes that will be very valuable for further research on temperature adaptation of plants have been evidenced.

**Electronic supplementary material:**

The online version of this article (doi:10.1186/s12870-016-0850-0) contains supplementary material, which is available to authorized users.

## Background

The grape berry is one of the most valuable horticultural crops with a total production of 7 10^9^ kg (http://faostat3.fao.org). Due to its various uses as fresh or dry fruit, wine and liquor, its economic impact is far greater than for other fleshy fruits, which are mainly consumed as fresh. Facing global warming with an expected rise of global surface temperature between 1.8 and 2.5 °C during the next century [1], many horticultural crops are already suffering from reduced productivity and altered composition which is likely to threaten global food supply [2–6].

High temperatures display various direct and indirect effects on the physiology of the grapevine fruit depending on the developmental stage. An accelerated malic acid breakdown [7, 8], a decrease in anthocyanins with possible variations in acylation in red-berry cultivars [9–12] and changes in the aromatic potential [13] are the most problematic consequences of elevated temperature on fruit quality. A rather moderate warming favored sugar concentration, which may lead to excessively alcoholic wines masking varietal aroma [13, 14] whereas extreme heat was reported to imped sugar accumulation and ripening [10, 15]. A shift of wine growing regions to higher altitudes or latitudes can be expected as a consequence of global warming [16] whereas traditional regions will not disappear [17] but might need varieties better adapted to elevated temperatures [18, 19]. New cultivars are thus needed to support a sustainable and more environmental-friendly viticulture in the long term. To develop tools for breeding programs, it is of utmost importance to understand the regulatory mechanisms underlying the response of grapevine fruit to temperature fluctuations.

Several genes have been directly related to thermotolerance in grapevines leaves [20] and fruits [21], still very few high throughput transcriptomic studies were performed to target global changes induced by temperature in fruits. These previous studies gave new insights in the possible adaptation of the berry to high temperatures, but they were limited to short heat stress [22] or to only one developmental stage during ripening [23]. In order to minimize biases and interferences in gene expression responses to one specific abiotic factor like temperature, other environmental conditions obviously need to be maintained as stable as possible. However the specific reproductive features and plant size of the grapevine makes experimental designs under strictly controlled conditions almost impossible. To circumvent these obstacles, the present study relies on microvine grown under controlled environments in small-scale climatic chambers. This new grapevine model is the Pinot Meunier L1 *Vvgai1* mutant [24] recently used for research on berry physiology [25], ecophysiology [26] and genetics [27]. This genotype is closely related to the PN40024 reference genome, facilitating the interpretation of RNAseq data.

The grapepevine berry displays a typicall double sigmoidal growth pattern that mostly results from the succession of two periods of vacuolar expansion [28] marked by a pronounced shift in the nature of prevalent osmoticums. During the green stage, proanthocyanidins are formed quite rapidely after berry set, quite simultaneously with cell divisions, and growth relies on the accumulation of 0.5 Eq of tartric and malic acid (pH 2.7) until a plateau is reached at lag phase. Ripening sets in with berry softening, the resumption of growth due to the onset of sugar accumulation, a simultaneous exponential decay of malic acid and accumulation of anthocyanin pigments [29–32]. A major transcriptomic reprogramming occurs during the abrupt drop in berry firmness that marks the transition between the lag phase and ripening, which is named véraison [33]. The heterogeneity in the timing of berry ripening within single clusters complexifies studies on berry development [34]. To evade biases introduced in gene expression by such asynchronous development, RNA-seq analyses were performed on homogeneous batches reconstituted after single berry biochemical analyses.

**Fig. 1:**
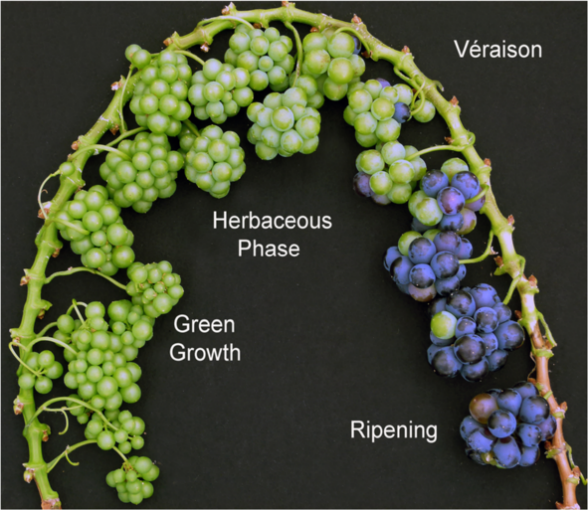
Microvine spatial fruit development. Leaves have been removed and main axis has been bent for illustration purposes

Daily fluctuating environmental conditions such as light and temperature as well as a molecular circadian clock are known to impact gene expression in plants and mammals [35–38]. Night transcriptomic profiling revealed many additional developmentally-regulated genes in addition to day regulated ones [25]. Circadian changes in genes expression were shown to be highly developmental stage-dependent, with very little transcripts exhibiting a continuous day-night pattern all along fruit development. Subsequently, it has been demonstrated that short heat stress triggered different transcriptomic responses depending on the photoperiod [22]. Further studies revealed similarities as well as important differences amongst daily gene regulation pattern in different cultivars [39]. Aware of these important advances, the present study on the effect of prolonged stress was conducted at day and night time on several berry developmental stages (Fig. 1).

In the present work, RNA-seq has been used to study the transcriptomic response to long-term temperature stress of grapevine fruits at five key developmental stages. Combining the use of the microvine model to be phenotyped in strictly controlled conditions (VPD, PAR, water supply) and an innovative sampling approach to circumvent berry heterogeneity, the study provides novel insights in temperature adaption of fleshly fruits.

## Results

### Temperature impact on berry physiology

**Fig. 2:**
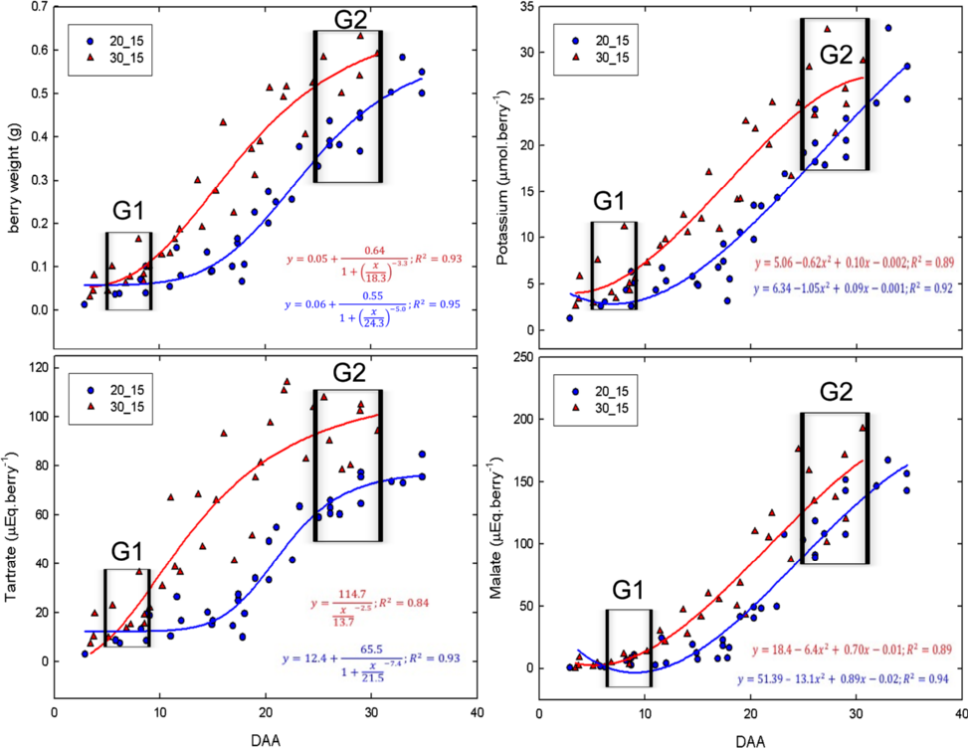
Kinetics of main berry solutes in green berries exposed to different temperature treatments. DAA : Re-calculated Days After Anthesis. Red: high day temperatures; Blue: low day temperature. Lines represent logistic regression fittings

Green berries were exposed to a similar night temperature of 15 °C whereas day temperature changed from 30 °C for hot to 20 °C for cold treatment (Fig. 2). Regarding the 30–15 °C regime, the experiment was stopped at 30 DAA (Days After Anthesis), when berry weight reached 0.6 g, close to the completion of the first growth phase which occurred at 0.64 g in preliminary experiments at 30–20 °C (Additional file 1: Figure S1). Tartrate synthesis ceased 20–25 DAA, reaching a plateau at ca 95 μEq.berry^−1^ in the 30–15 °C treatment. Malate content reached 150–200 μEq.berry^−1^at 30 DAA. The temperature drop of 10 °C during the day delayed berry growth and the accumulation of the three major osmolytes by 5.5 days, without affecting their maximal accumulation rates (Fig. 2).

To analyze the impact of temperature on the fruit transcriptome during green phase, two stages were selected during the first growth phase, sampled two hours before the onset of day and night, respectively. Stage Green 1 (G1) was selected right after fruit set, when intense cell division occurs simultaneously with tartaric acid and proanthocyanidins accumulation, and stage Green 2 (G2; Fig. 2) corresponded to the late-green phase, when tartaric acid synthesis was nearly complete, but before the cessation of growth and malic acid accumulation in lag phase.

**Fig. 3:**
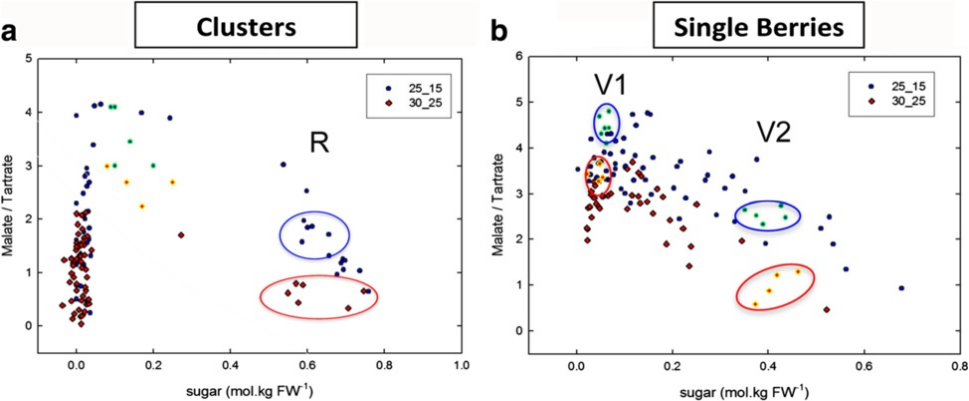
Malic acid (MA)/Tartaric acid (TA) ratio as a function of sugar concentration for high (red) and low (blue) temperatures. **a** represents the average composition of clusters along the proleptic axe. **b** represents single berry analysis from the clusters highlighted in green or yellow in A, each point represents a berry. Highlighted points represent berries chosen for RNA-seq

**Fig. 4:**
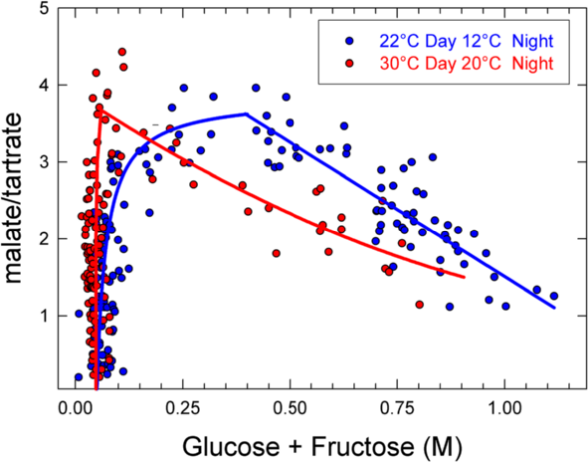
Malate accumulation following the onset of ripening in transgressively cool conditions. Each point represents one cluster (~30 berries). At the end of each treatment, all clusters were harvested simultaneously on 5 plants. The induction of ripening is marked by the simultaneous inductions of malate breakdown and massive hexose storage in berries at 30–20 °C, as typically observed in vineyard conditions. The onset of malate breakdown is delayed to 0.4–0.5 M hexoses following a 3 months growth period at 22–12 °C

During the second growth period known as ripening, higher temperature gradients (25/15 °C and 30/25 °C; day/night) were applied in order to mimic the temperature increase that occurs during the summer. Furthermore, it is known that night temperatures during ripening noticeably impact final berry composition [9, 40]. In Fig. 3a, the sugar concentration of average clusters is plotted against the malate/tartrate (MA/TA) ratio. Tartaric acid per berries was unaffected by temperature (Table 1). The breakdown of malic acid began when sugar accumulation started, and it was more pronounced under higher temperatures, as widely described in the literature [41, 42]. This typical pattern was nevertheless strikingly modified in transgressively cool conditions, since the synthesis of malic acid unambiguously persisted following the induction of sugar storage at 22–12 °C, its breakdown being delayed untill 0.4–0.5 M hexoses were accumulated (Fig. 4). Such uncoupling of malate breakdown from the onset of sugar storage has never been reported before. Noticeably, base 10 Growing Degree Days was doubled between the cold and hot conditions in this last experiment, without marked departure in the timing of the onset of sugar storage (veraison) (typically : 45–50 Julian days after anthesis, see Additional file 1: Figure S1).

**Table 1:** Biochemical analysis of post-véraison berry batches selected for RNA-seq analysis

Sample	Berry weight (g)	Malate (μEq.berry^−1^)	Tartrate (μEq.berry^−1^)	Sugar (mg.g FW^−1^)
V1_cold	0.9 ± 0.2	635 ± 43	149 ± 13	0.11 ± 0.02
V1_hot	1.0 ± 0.1	649 ± 77	160 ± 9	0.12 ± 0.01
V2_cold	0.9 ± 0.1	354 ± 80	138 ± 24	0.43 ± 0.01
V2_hot	0.8 ± 0.1	155 ± 94	141 ± 34	0.4 ± 0.02
RDay_cold	1.5 ± 0.1	230 ± 25	147 ± 27	0.6 ± 0.04
RDay_hot	1.4 ± 0.25	85 ± 20	139 ± 22	0.57 ± 0.02
RNight_cold	1.4 ± 0.2	235 ± 45	130 ± 22	0.62 ± 0.03
RNight_hot	1.55 ± 0.15	79 ± 40	140 ± 14	0.66 ± 0.08

To reduce biases introduced by the asynchrony in the onset of berry ripening [34], individual berries were analyzed separately in clusters displaying first signs of véraison (either softening or very initial coloration of one berry at least) (Fig. 3). Based on sugar concentration, the distribution of individual berries (Fig. 3b) inside these clusters (Fig. 3a) displayed virtually all developmental stages from mid green phase to mid-ripening, `regardless of their coloration, which was still green for most of them. Two batches of homogeneous berries were reconstituted based on their sugar and organic acid content for further RNAseq analysis (V1: Véraison 1 and V2: Véraison 2; Fig. 3b). Average clusters, where all berries were fully colored were then selected for ripe stage (highlighted in Fig. 3a: R-Ripening), taking advantage of the grouping of berries during late ripening [34].

Biochemical analysis (Table 1) confirmed the fast evolution of berries between the two temporarily very close stages V1 and V2, with the same berry weight but a 4 times higher sugar content in V2 than in V1. Sugar accumulation just started in V1, before malate breakdown could be observed. 50 % of malate was respired in V2 in the cold, increasing to 75 % in the hot. From V2 to R, the sugar concentration increased by 30 % only, but it was accompanied by a 70 % increase in berry weight. This thus seems as if the apoplasmic pathway of sugar loading [90] was fully activated after V1, and closer to V2. The decrease in malate was as expected quicker under warm temperatures with another 50 % drop as compared to a 35 % under cold conditions. Notably it was not as quick as between V1hot and V2hot. The tartrate content per berry is invariant from V to R and from hot to cold (Table 1), which validates the use of the dimentionless malate/tartrate ratio as a proxy for malate per berry in Figs. 3 and 4. It can then be concluded from Fig. 3b that berries at V1 have just started to accumulate sugars but that malate breakdown has not started yet. In V2, with 0.4 M hexoses, sugar loading certainly occurs at maximal speed (see Additional file 1: Figure S1). Malate breakdown (net flux) has started in V2 cold, but is close to completion in V2 hot. The situation is not fundamentally different in R, except that malate has reached its lower limit in hot.

### Temperature impact on the berry transcriptome

#### Overview of heat induced transcriptomic changes

A total of 674 millions reads have been sequenced from all selected berry stages exposed to different temperature regimes and sampled during the day or at night. Following filtration and alignment with the CRIBI unigene set, analysis of differential expression between the two temperature conditions at each stage and time point yielded in a total of 10 787 differentially expressed genes (DEG; padj < 0.05, fold change (fc) > 2; Additional file 2: Table S1). Hierarchical clustering performed on normalized counts including all biological triplicates confirmed consistency of stage selection and temperature conditions (Additional file 3: Figure S2). In this analysis, day and night conditions can only be clearly distinguished under high temperatures in green stages, whereas they are less well separated under cold temperatures. Interestingly, the reconstituted first véraison stage in cold conditions (V1cold) was more different from V2cold than from V1hot and V2hot and the closest one to the green berry, confirmed by principal component analysis (Additional file 4: Figure S3). In the first PCA (Additional file 4: Figure S3A), where the first two PCs account for 92 % variance and explain mainly development, daytime and temperature induced changes cannot be distinguished. However all four véraison conditions are very well separated from the others. This highlights the transcriptomic shift occurring at véraison [33] and the importance of a precise distinction of berry stages notably during this decisive and abrupt transition period. Temperature regime can be distinguished on PC 3 (Additional file 4: Figure S3B). Temperature seemed to have induced more important changes in green stages than in ripening stages. Stage separation between G1 and G2 was more obvious under hot condition and this was inversed on previous short heat stress studies [22]. Puzzlingly, this was still observed two hours after the stress has ceased when plants were maintained in rather cool conditions (15 °C), at night.

**Fig. 5:**
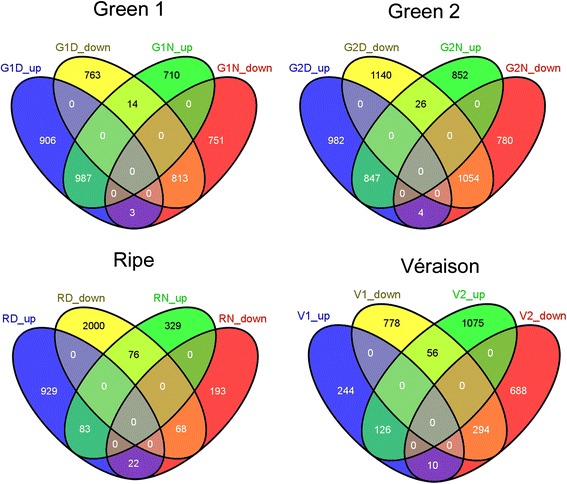
Venn diagram of heat modulated transcripts (fc > 2, padj. < 0.05) comparison for day and night separately depicted for green (G1 and G2) and ripening stages (R). For véraison stage where only day sampling was performed heat modulation is compared between V1 and V2

The number of heat modulated transcripts (lfc > 1, padj < 0.05; Fig. 5); Additional File 12 : Figure S7 greatly changed according to berry developmental stage and daytime. The total number of heat-induced transcripts at day was similar in both green stages, with 1896 G1D_up and 1833 G2D_up, half of them being still induced at night, whenever the temperature gradient was collapsed. In this respect, additional 710 G1N_up and 852 G2 N_up were only detected at night. A similar distribution can be observed regarding heat repressed genes in green berries, with 1576 G1D_down, with half of them still repressed at night, plus an additional set of 751 specifically repressed at night (G1N_down). In G2, as much as 2195 genes were repressed at day (G2D_down) therein 50 % were down-regulated at night, with an additional 780, which only responded at night (G1N_down). This pattern changes at the ripe stage (R), where only a small number of genes were commonly heat modulated at day and at night. Remarkably, in ripening berries (R) a high number of genes (98) even inversed their temperature response from day to night (Additional file 5: Figure S4). Due to the limited number of berries at véraison, only day sampling was conducted in V1 and V2, with 3.4 times more heat induced genes in advanced berries (V2). A similar tendency was observed in short heat stress studies where the more advanced post-véraison berries showed a larger number of heat modulated genes [22]. A global pageman analysis of enriched functional categories has been conducted for all heat-modulated transcripts in all conditions (Additional file 6: Figure S5A-C). Additionally, functional categories of transcripts modulated only at one stage and time point upon heat were analysed separately for green stages and ripening (Additional file 7: Figure S6). Within temperature-induced genes heat shock related functional categories (protein folding, chaperon mediated protein folding, HSP-mediated protein folding; Additional file 6: Figure S5B) were significantly enriched in most developmental stages at day and night. Whereas primary and secondary metabolisms such as amino acid synthesis (Additional file 6: Figure S5B) and phenylpropanoid related pathways were mainly repressed in advanced green berries (Additional file 6: Figure S5C).

**Fig. 6:**
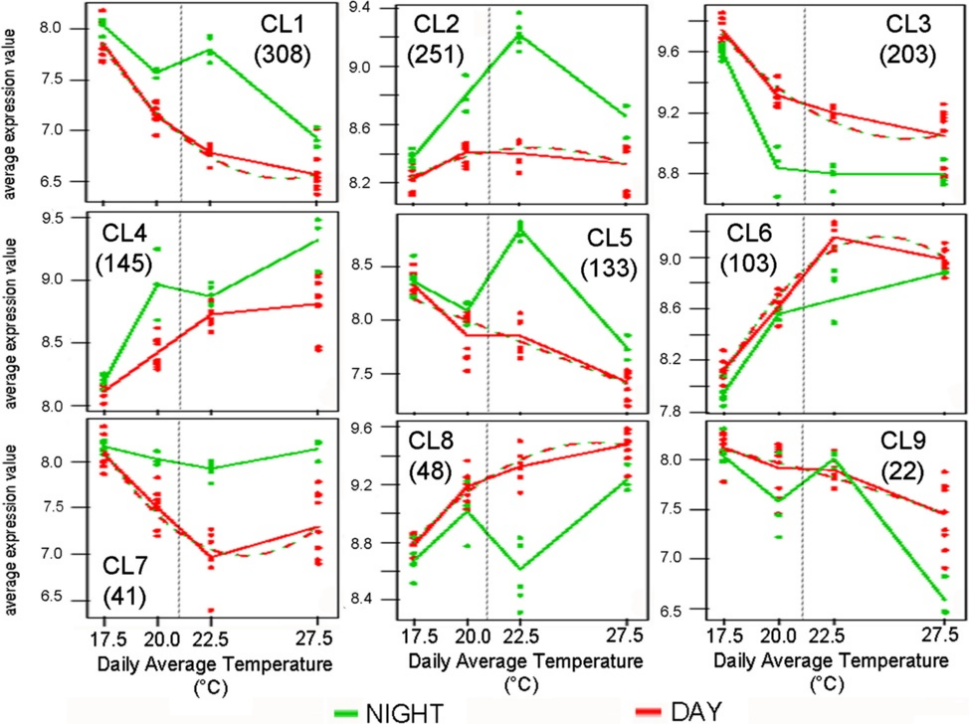
Clusters of genes differentially modulated by temperature at day or nighttime, identified by time-series analysis. Name of cluster with the corresponding number of allocated genes is depicted within each graph. Abscissa corresponds to the calculated daily mean temperature of each treatment. Dotted vertical line separates pre-véraison (Green) from post-véraison (Ripening) stages

To decipher day/night specific gene modulation, a two-class maSigPro time series analysis [43] was conducted treating daily mean temperature as quantitative variable and day and night as qualitative variables. Thereby 1254 DEGs (padj < 0.05) could be identified and allocated to nine clusters by hierarchical clustering (Fig. 6). In this way, transcripts whose temperature response diverged significantly at day or night could be detected (Additional file 8: Table S2). Since average temperatures changed as well from green (17.5 and 20 °C) to ripening phases (22.5 and 27.5 °C) a developmental stage effect is as well detected this way.

Considering that night temperatures were maintained for green berry treatments (17.5 & 20 °C) the somehow opposed clusters Cl3 and Cl4 as well as Cl2 and Cl9 are particularly interesting since their day/night differences are very pronounced during these phases.

Significant functional enrichment (padj < 0.05) was only detected in two clusters (Cl2 & Cl3). In cluster 2, which showed a stable pattern along different temperatures at day but a strong increase at night up to 22.5 °C, enriched categories were related to Regulation of genes expression and Transcription factors. TFs included a trehlix transcription factor (VIT_00s0204g00020) that binds light regulated genes and would be putatively involved in salt and pathogen stresses [44].

**Fig. 7:**
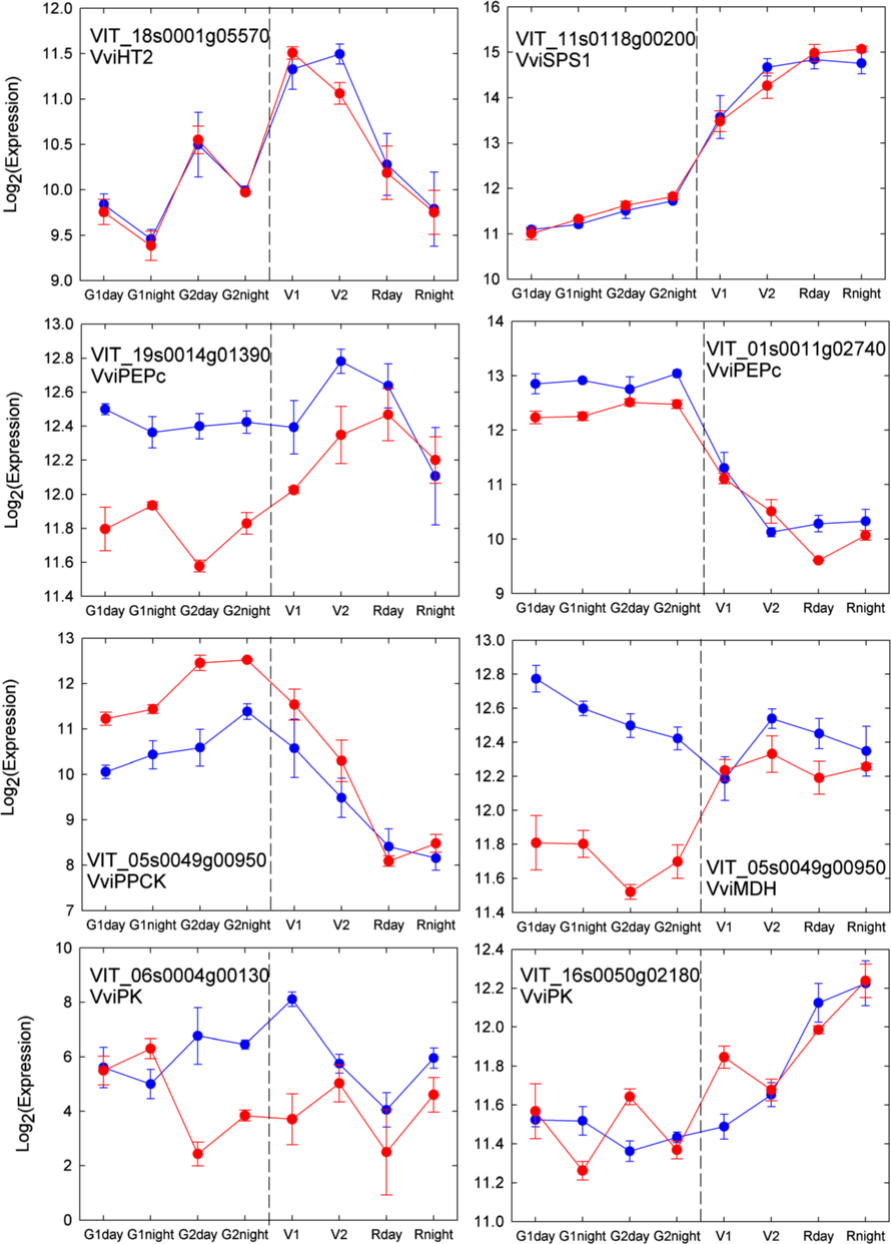
Expression pattern of sugar and acid related transcripts under different temperatures

In addition to chaperone/HSP mediated protein folding, primary metabolism was overrepresented in Cl3, more precisely respiratory chain phosphorylation, and transport overview, with the noticeable exemples of AHA10 and AHA4 H^+^ATPases (VIT_09s0002g00130, VIT_15s0048g00170, Fig. 7). Several putative transcription factors (TFs) were allocated to cluster 4. A TCP family TF (TCP 11 (VIT_08s0040g01600) involved in growth, cell proliferation, and organ identity in plants circadian regulation in Arabidopsis thaliana [45], a Scarecrow TF (TF14; VIT_06s0004g04980) and a PLATZ transcription factor (VIT_16s0039g01560).

## Discussion

### Heat shock response decreases during ripening

A very important strategy of plant adaptation to high temperatures is the production of heat shock proteins (HSPs). They act mainly as molecular chaperones, which are essential for maintenance and/or restoration of protein homeostasis [46, 47]. HSPs participate in protein folding, assembly, translocation and degradation in many cellular processes and can thereby prevent denaturation and preserve stability and function of proteins under abiotic stress [48]. HSPs are crucial for thermotolerance [20, 49, 50] but also for tolerance to other abiotic stresses such as salinity or drought [51]. They can be organized into Hsp 100, Hsp 90, Hsp70, Hsp 60 and small HSPs [52] and are encoded by heat shock genes (HSG) [53].

The enrichment of heat stress related functional categories (Additional file 7: Figure S6) is in agreement with previous temperature studies conducted on green and ripening grapevine fruits [22, 23]. Unexpectedly, the over-representation of these categories was not detectable in V1 and was even inversed in ripening berries at night (RN). The enrichment is mainly due to the expression profiles of several isogenes coding for small heat shock proteins (HSP kDa 17.6: VIT_13s0019g02770, VIT_19s0085g01050 VIT_13s0019g02780: Cl 6) repressed by heat in RN as well as two heat shock transcription factors coding genes, HSF A3 (VIT_08s0007g03900) and VvHSF A2 (VIT_04s0008g01110).

According to the literature, HSP 90′s play an important role in plant development, stress response, and disease resistance [54]. In human, their key role in controlling normal cell growth and in promoting tumor cell development as been recently put in evidence [55]. In grapevine fruits, a HSP 90 transcript (VIT_16s0050g01150) showed a very high temperature induction except for V1, V2 and RN.

Hsp70s are encoded by a multigenic family of 14 genes in *Arabidopsis thaliana* and 12 in spinach [56]. Several Hsp70 coding isogenes showed opposed expression in response to temperature in present study. E.g. VIT_13s0019g01430, VIT_16s0098g01580 and VIT_02s0025g02140 were repressed by heat in particular in green berries and allocated to cluster 3. As opposed to VIT_05s0020g03330, VIT_14s0060g02340 and VIT_06s0004g04510 (Cl6) that exhibited most pronounced induction in G2 berries. Interestingly one HSP 70 transcript (VIT_08s0007g00130) showed a very high night specificity where it was repressed in all stages but even up-regulated at day in R. Amongst all HSPs a small HSP 22 kDa (VIT_18s0089g01270) showed the highest induction with a 128 fold increase in the green berry (G2) but no, or quite limited induction in V1 and thereafter. This unambiguous expression pattern of HSP genes is known from other plants such as *Arabidopsis thaliana* [56] where most HSPs showed a very rapid induction within 30 min to 2 h of heat stress (37 to 45 °C). However, other authors even observed an induction of genes belonging to this family in response to cold [57]. An adaptation was also observed in grapevine leaves exposed to heat stress (45 °C, 5.5 h) with several heat shock genes being induced upon stress but recovered if not down-regulated rapidly within 20 h post-treatment [58].

HSG transcription is controlled by heat shock transcription factors (HSFs) [53, 59, 60] that can be classified in three groups according to their structural characteristics and phylogenic comparisons [61]. In this respect, *Arabidopsis thaliana* includes 15 HSF A, 5 HSF B and one HSF C [48].

As for HSP, the expression of HSFs in response to temperature showed diverse patterns amongst stages and time. E.g., all members of the HSF B group showed consistent repression two hours after the interruption of the hot temperature treatment at night in green berries. The repression was just beyond the significance threshold in G2 for HSF B4 (VIT_06s0009g02730) and HSF B2A (VIT_10s0597g00050). A HSF B2B encoding transcript (VIT_02s0025g04170), which is involved in pathogen resistance in *Arabidopsis thaliana* [62] was down-regulated at night in G1 and G2. The functions of class B HSFs are not very clear but indications for their role in the repression of HS gene expression during recovery and in pathogen resistance have been reported in several studies [48]. The latter HSFs already displayed night specificity in short heat stress studies (37 °C; 2 h) [22], where they were heat induced at night in green and ripening berries. However, this apparent regulation may have alternatively resulted from the greater temperature gradient imposed at night. No long-term temperature stress is applied at night in green berries in the present study, but they were cooled by 15 °C at sunset, 2 h before sampling, in the hot treatment. Then, HSF B family members apparently sense short but intense temperature gradients at night, in green stage, what would be appropriate to confirm on exhaustive kinetics studies.

Members of the HSF A family and one HSF C1 (VIT_11s0016g03940) showed up-regulation tendencies in day treatments. Whereas induction of HSF A family members is only punctual with a HSF A3 (VIT_08s0007g03900) in G1 and Rnight, HSF A6B (VIT_00s0179g00150) induced in G1 and *VvHSF A2* (VIT*_04s0008g01110*) only in ripening berries, curiously both of them showed a repression in RN. The latter was amongst the most induced HSFs in tomato, *Arabidopsis thaliana* and rice [63, 64] and is thought to regulate general stress-related genes such as *GolS1* (*galactinol synthase 1*) and *APX* (*Ascorbate peroxidase 2*) [48]. *VvHSFA2* was heat induced in ripening berries from fruiting cuttings and showed a maximum induction at 8 h of treatment, subsequently decreasing to a minimum after 21 days [21]. This fast heat response was confirmed in short heat stress studies where an up to 32 fold induction was detected in all berry stages [22].

Putatively regulated by *VvHSFA2*, *VvGolS1* [21, 48] represents the key enzyme in the synthesis of galactinol and other raffinose family oligosaccharides that accumulate as osmoprotectants in plant cells after heat stress [66]. In the present study *VvGolS1* (VIT_07s0005g01970) was equally activated day and night in green berries but 4 times more in G2 than in G1, which represented a marked de-correlation from *VvHSFA2* thus indicating that *VvHSFA2* does not solely regulate it.

A transcription factor (*MBF1c*), acting upstream to salicylic acid, ethylene and trehalose has been shown to be involved in the heat stress response of *Arabidopsis thaliana* [67, 68]. *VvMBF1c* (VIT_11s0016g04080) showed a higher induction at night than at day in green berries, following short heat stress [22]. In the present study, its temperature induction was found limited to green stages with a slightly higher induction at night as well, two hours after both treatments reached the same night temperature. Another member of this family, MBF1a (VIT_19s0014g01260) showed an induction only during daytime indicating a more direct heat response or a different photoperiod regulatory mechanism.

The multifacetted regulation of HSFs and HSPs along berry development confirms the importance of heat adaptation within fruits facing high temperatures. Short and long term adaptive regulations exist within the heat shock family where some do not show any long-term temperature adaption and keep on responding to daily temperature fluctuation on a real time basis. The most extreme ones are down-regulated once the stress ceased, exactly as if the fine-tuning of protein abundance vs temperature was more determining than that of transcript level. Others are consistently up-regulated under warm regimes without respect to daily temperature fluctuation or circadian changes even at constant night temperatures.

Surprisingly, at the onset of ripening, HSG transcription seemed to be less responsive to temperature. The present PCA and HSG expression data suggest both that the green berry is intrinsically more susceptible to heat than the ripening one. As a matter of fact, the temperature modulation is overwhelemed by the drastic transcriptomic reprogramming associated with ripening (ie HSP22A, MBF1C). Such a developmental regulation of HSG anticipates the usual variation of seasonal temperatures along fruit development, since green stage occurs from end of May till mid July in North hemisphere, when daily temperatures are still moderate. The onset of ripening occurs from mid July (Languedoc) till mid August (Germany) and thus falls in the warmest period of the year. Moreover, in contrast with green fruits, whose vacuolar compartmentation can cope with energy shortage, what gives time for an adaptative answer, the hazard of cytosolic acidosis due to stress dramatically increases with the permeability of the vacuolar membrane after véraison [67]. The stability of H^+^-pyrophosphatase up to 65 °C highlights how vital is the continuation of the energization of the tonoplast during thermal stress [68]. This adaptation would be vain if cellular energy was massively drained by *de novo* synthesis of HSP.

### Cold temperature delays berry development in the early stages of development

In previous studies, temperature effect on berry growth was not unambiguous, as it was the case in the present study. Drawing upon previous studies in general green berry development seems to be accelerated by temperature until a certain threshold, which appears to depend on the cultivar. In Cabernet Sauvignon treated 2–5 days before and 12–18 days after bloom, berry growth displayed the optimal temperature of 35 °C and then declined [69]. Other studies didn’t detected any changes when temperature gradients were applied during the whole development [8] or post-véraison [70]. Matsui et al., 1986 [71] reported a growth inhibition in Thompson Seedless and Napa Gamay berries treated at 40 °C for four days during green stage. Similarly, a reduction in berry volume and fresh weight was observed on Semillon during heat waves [10, 15]. Vice Versa a decrease of cell division has been reported below 15 °C during the green growth period [69]. A hastening of fruit development due to heat was also described in other fruits such as apple [72] and tomato [73].

In the present study, several transcripts coding for genes that have been previously linked to pericarp development or fruit growth have been up-regulated by high temperature in green berries. Their expression pattern is summarized in Additional file 9: Figure S8. Amongst them, was a wrky transcription factor (VIT_17s0000g05810), which is thought to regulate skin and flesh ripening [74, 75]. This TF correlated with the faster development of green berries under hot condition. The same transcript was strongly down-regulated in ripening berries, which questions its function during late stages of fruit development. A cytochrome P450 78A homologue (VIT_17s0000g05110), a gene partly controlling fruit weight in *Solanum lycopersicum* L. [76] was induced by temperature in G1. Several other genes in relation with berry growth were also found heat induced, such as VIT_18s0001g13930, which was repressed in the fleshless mutant unable to differentiate from the L2 layer the highly vacuolated cells that are characteristic of the mesocarp tissue [77]. Other functions related to cell expansion are likely responsible for the changes in berry weight, in the first place the increase in cell wall area. Schlosser et al., [78] showed that a cohort of candidate cell wall-modifying enzymes (*expansin, glycosyl hydrolase, pectinesterase, pectate lyase, cellulose, XET-xyloglucan endo-transglycosylase*) was highly expressed during berry growth. Expansins are key regulators of cell wall extension [79, 80]. Positive correlations between the presence of expansin activity, epitopes or transcripts and growth rates have been observed in several plants [81]. Here, a beta-expansin coding transcript (Exp 4: VIT_15s0021g02700*;* Additional file 9: Figure S8) was highly up-regulated under warm conditions very early in green berry development (G1) and not during later stages, whereas an Exp 2 (VIT_13s0067g02930) was heat induced in all green stages. These expression patterns suggest temperature accelerated cell growth and wall extension in green berries, which is supported by expression pattern of *cellulose synthase* (e.g. VIT_05s0049g0050; Additional file 9: Figure S8). Genes of the latter superfamily are among the most important players in the biosynthesis of plant cell walls, which are composed of biopolymers such as celluloses, hemicelluloses, pectins and lignins [82]. Water import, mediated by MIP and PIP genes, is also essential for cell expansion [84, 85]. Still aquaporin’s coding transcripts such as a PIP2.2 (VIT_13s0019g04280) have been identified as candidate genes in several studies on the determinism of berry weight and cellular expansion [74, 86]. Here, no correlation could be observed with the previous candidate genes, nor could the temperature response of a further aquaporin (VvSIP1) [87] be confirmed.

Interestingly, the hasting of berry growth under warm temperature seemed to be mainly related to enhanced cell growth, not to increased cell division. The enrichment of heat-repressed transcripts in DNA metabolism related functional categories (Additional file 6: Figure S5) rather points in favor of a decreased cell division rate. The expression patterns of several genes involved in DNA replication support this hypothesis. Cell division cycle proteins (CDC) interact in the MCM (mini-chromosome maintenance) complex and play a key-role in the regulation and elongation stages of eukaryotic chromosomal DNA replication [83, 84]. Two CDC7 (VIT_15s0021g01380, VIT_00s0616g00030) and CDC45 transcripts (VIT_12s0142g00280) showed down-regulation by high temperature in green berries, and were also co-regulated with DNA Helicase (VIT_16s0013g00300) and a centromere protein (VIT_00s0313g00010).

Present molecular data suggests that those genes play an important role in early berry development since they correlate with the hastening of early berry development.

### Temperature impact on primary metabolism – cold de-correlates malic acid breakdown from sugar storage

Cool temperatures during the growing cycle of the vine result in higher malic acid content in ripe berries as the consequence of a slowing down of post-véraison malic acid breakdown [7, 8, 23, 85–87]. Few temperature studies also showed that temperature would promote the synthesis of malic acid synthesis before véraison [87, 88]. However, since a strict selection of post-véraison berries has never been performed before, these studies suffered from a statistical bias that led to underestimate the velocities of malate breakdown and sugar storage. Average ripening time, commonly observed to occur within 40 to 60 days after veraison [30, 31] would last no more than 15 days at berry level [29]. Single berry analysis (Fig. 3) clearly showed that within a grapevine cluster a high number of berries may still be green and acidic whereas others fruits have reached mid final hexose concentration (around 1 to 1.1 M hexose are expected in ripe stage). Moreover, the onset of malate breakdown is strongly delayed at 22–12 °C, in constantly cool culture conditions that have never been applied hitherto to grapevines during the ripening period.

**Fig. 8:**
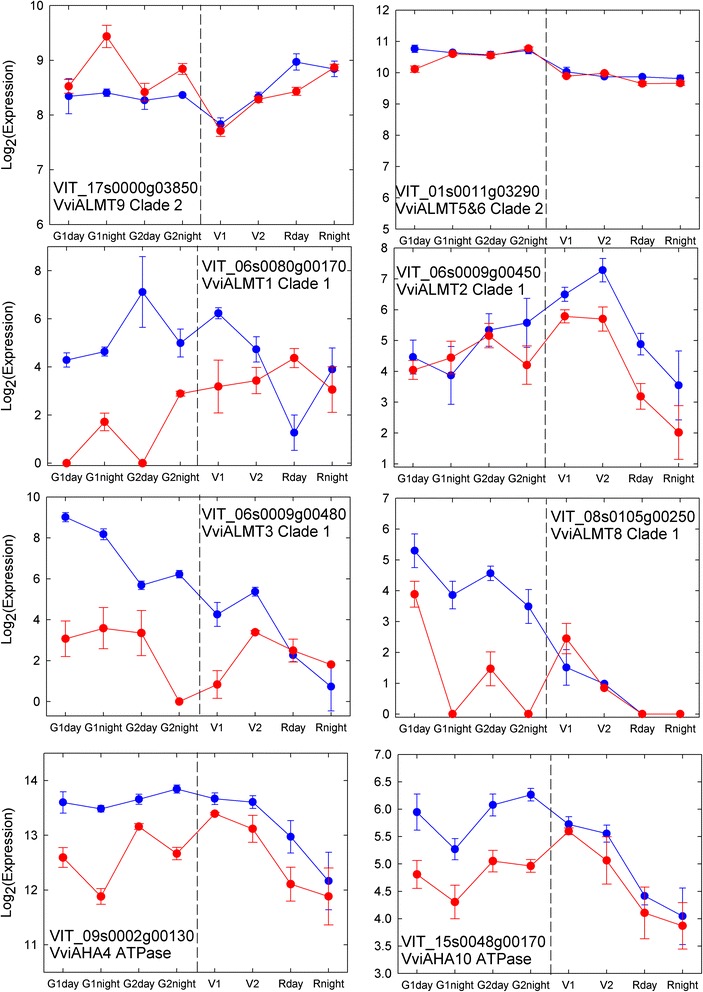
Expression profiles of malate transporters and selected H^+^-ATPases

Genes encoding most enzymes of primary metabolism remained noticeably stable with temperature, even those exhibiting some degree of developmental or day-night regulation, like VviHT2 (VIT_18s0001g05570) or Sucrosephosphate synthase 1 (VviSPS1; VIT_11s0118g0020; Fig. 6). The first step of MA (malic acid) synthesis involves the carboxylation of PEP by PEPC (*Phosphoenolpyruvate carboxylase*) and yields in OAA (oxaloacetate). OAA is further reduced to MA by MDH (*Malate dehydrogenase*) or supplied to the TCA cycle [41]. The expression patterns of specific members inside these multigenic families (Fig. 7) suggests an enhanced MA synthesis under cool temperatures which starts delayed (Fig. 2). This is in agreement with previous studies on the corresponding enzyme activities in green [85] and ripening berries [23, 85] as well. Moreover, a *Phosphoenolpyruvate carboxylase kinase* (PPCK) gene displayed increased expression under warm temperatures, which indicates that post-transcriptional regulation of PEPcase through phosphorylation makes it less prone to feed-back inhibition by cytosolic malate, at elevated temperature. The expression of a *Pyruvate kinase* (PK) gene (VIT_06s0004g00130) was strongly impaired by temperature after G1 in all samples showing reduced MA content (Fig. 7), which points in the same direction as in the quoted study and supports the hypothesis of a reduced glycolytic flux and an enhanced anaplerotic capacity of the TCA cycle at the expense of malate, in heated fruit. In plants, PK is thought to provide bottom-up control of plant glycolytic flux from hexose-phosphates to pyruvate owed to PEP’s pronounced feedback allosteric inhibition of ATP- and PPi-dependent phosphofructokinases. The PK isogene VIT_16s0050g02180 showed a very distinct day night modulation with heat repression tendencies at night and vice versa during the day. Interestingly, the corresponding enzyme activity was also reduced under high temperatures but only at night [85]. The expression of malic enzyme was not significantly modulated upon heat (data not shown), nor was the case regarding its enzymatic activity [85]. VviALMT9 (aluminium activated malate transporter 9; VIT_17s0000g03850.1, see Fig. 8) is the sole malate (and tartrate) transporter which function at grapevine tonoplast has been documented so far [86]. Increased expression during ripening makes VviALMT9 a privileged candidate gene regarding the futile recycling of malate that takes place at the tonoplast simultaneously with malate breakdown [68, 86]. However, Fig. 7 unambiguously shows that this inward-rectifying channel is already expressed during the tartrate and malate accumulation period, well before the first sample characterized in [86] (49 DAF, close to V1). The weak but significant modulation of VviALMT9 expression by temperature would favor malate accumulation in green stage (Fig. 7). Moreover, VviALMT9 appears preferentially expressed during the nocturnal fruit expansion period in green stage, giving some support to the previous hypothesis of differential day-night regulation of malic acid metabolism [86]. Phylogenetic analysis clearly suggests that additional clade II ALMT should be targeted to berry vacuolar membranes (Additional file 10: Figure S9), like VviALMT5&6 in VIT_01s0011g03290, as the best orthologs of MDP24429 and MDP252114 genes at the Ma1 locus in apple, with the last one encoding a truncated ORF in cultivars of low acidity [87]. In fact, Vvi-ALMT9 could rather be the ortholog of At3g18440, the chloride channel gated by malate that controls stomatal aperture in *Arabidopsis* [88]. Whatever, none ALMT sharply emerged from clade II, regarding the effects of development or temperature. By contrast, many clade I ALMTs displayed considerable regulation (ie VviALMT1, 2, 3 & 8, Fig. 7), when compared to previous genes in the malate pathways. AHA4 and AHA10 H^+^ATPases, that may regulate cytoplasmic and apoplastic pH in conjunction with ALMTs, also share with many ALMT consistant repression by temperature and ripening, in addition to significant day-night dependance (Fig. 7). Multifacetted changes in expression of these primary and secondary transporters likely addressed to the plasma membrane [88] may tightly regulate malate homeostasis in berry apoplast [89], in particular when water import shifts from xylem to apoplastic phloem unloading at véraison [90]. This highlight apoplastic malate shuttles as possible regulatory targets for berry development and adaptation, acting between malate storage pool in the interior flesh and peripheral areas where malate is dissimilated [91]. Alternatively, AHA10 orthologs are recruited for proper vacuolar compartmentation of organic acids, anthocyanin or proanthocyanidins in, respectively, Citrus fruits, Petunia petals and Arabidopsis seeds [65, 92, 93]. The vacuolar targetting of this ATPase of low stochiometric ratio even provides a thermodynamical basis to the acidification of citrus juice vesicles below pH 2.5 [94]. Instead than a contamination as initially believed, AHA10 vanadate sensitive activity would represent 30 % total vacuolar ATPase activity in ripe berries [65] and certainly more in young berries, owing to its expression profile. Present results are then consistant with a strong compartmentation control of berry acidity, but further work needs to be done regarding the tissue and membrane specificity of these ALMTs and AHA othologs in berries. The so far unprecedented observation of an uncoupling of malic acid respiration and sugar accumulation under cool temperatures (22–12 °C) indicates that, at the onset of ripening, the grapevine is somehow not obliged to consume malic acid in lieu of sugar, nor utilize it as substrate for neoglucogenesis. This observation could be explained by a better carbon status of plants exposed to cool conditions, as confirmed by direct measurements of carbohydrates in the canopy (Luchaire *et al*., Spatio-Temporal Analyses of Microvine Reveal an Uncoupling of Growth and Sugar Storage under Climate Warming; submitted to American Journal of Enology and Viticulture). It gives independent support to the concept that vacuolar sugar storage turns so efficient during ripening that cytoplasmic sugar becomes limiting [146], hence the induction of malate respiration, neoglucogenesis and futile hexose recycling, excepted in rare situations where the balance between fruit respiratory demand and the import of photoassimilates becomes particularly favorable (inhibition of night respiration at low temperature).

### High temperature repressed secondary metabolism related transcripts

Phenolics are defense compounds acting as repellent against herbivores and protect leaves from photo damage by acting as antioxidants [96]. They principally consist of flavonoids, which can be grouped into three classes: flavonols, anthocyanidins and proanthocyanidins (or condensed tannins). Their interaction with biotic and abiotic environments is difficult to estimate but they play an important role in plant development under constraints [97]. They constitute major wine quality determining substances and are largely responsible for color and astringency as well as for health benefits attributed to wine consumption [98]. Phenolic compounds derive from the phenylpropanoid pathway which was shown to be repressed by short heat stress in ripening grapevine berries [22]. Many isogenes coding for the key enzyme of the phenylpropanoid pathway, *phenylalanine-ammonia-lyase (PAL),* were also found repressed by high temperature in the present study differentially according to developmental stage and photoperiod. Several *PAL* isogenes were specifically repressed during the day on ripening berries, and down-regulated up to 32 fold (VIT_16s0039g01320, VIT_16s0039g01240, VIT_16s0039g01360). Four PAL isogenes (VIT_08s0040g01710, VIT_06s0004g02620, VIT_16s0039g01300, VIT_13s0019g04460) showed a heat repression in the G2 day as well at night. Isogenes coding for *chalcone synthase* [99] as the first committed enzyme in flavonoid biosynthesis [100] exhibited a similar pattern in green berries where it was repressed in G1 day and night (VIT_14s0068g00930) and a second CHS coding isogene (VIT_14s0068g00920) was repressed in G2 day and night. Interestingly, in short heat stress treatments [22] a modulation of this enzyme was only observed in post-veraison berries. Several *flavanone-3-hydroxylase* coding genes (*F3H;* VIT_04s0023g03370*,* VIT_18s0001g14310, VIT_06s0009g02970), which mediate the addition of hydroxyl groups to the B ring of flavones, dihydroflavonols, and flavonols [101] were heat repressed in green berries. They act upstream to *flavonol synthase* [102] required for flavonol biosynthesis [103], which showed down-regulation as well (VIT_13s0106g00550, VIT_13s0047g00210: Cl3).

Abscisic acid [76] is thought to control the accumulation of flavonoids through the transcriptional control of enzyme activities in their biosynthetic pathways [104]. ABA levels are highest in young green berries and decrease towards to lag phase to resume accumulation at the onset of the ripening stage [105, 147]. In the present study, one transcript coding for a key enzyme in ABA synthesis (*NCED: nine-cis-epoxycarotenoid dioxygenase)* was found to be repressed by temperature in green berries, the same gene was found to be down-regulated in berries of water stressed vines [106]. Similar trends were observed on apples where low temperature and drought conditions increased endogenous ABA [107].

The repression pattern of the latter transcripts illustrates an impairment of the phenylpropanoid pathway by elevated temperature, notably of flavonoid biosynthesis, and confirms previous studies on grapevine [108]. Interestingly, several *PAL* and *CHS* isogenes repressed by heat during the day were still repressed at night, two hours after the cessation of the temperature treatment in green berries (15 °C). This suggests that the global long-term temperature perception of plant prevails over nycthemeral fluctuations in the regulation of this pathway.

### Genes involved in tannin synthesis in the developing green berry are favored by cool temperatures

The phenylpropanoid and flavonoids pathways are integral to proanthocyanidins (PA) formation: they are oligo- and polymers of flavan-3-ols and involved in various physiological and biochemical processes; they possess herbivore deterrent and antifungal properties [109–111]. They are often referred to as condensed tannins and contribute to astringency and color stability in wine [112]. Monomeric, oligomeric, and polymeric flavan-3-ols are found in high concentrations in skins and seed tissues of grapevines [113] while in the mesocarp they are highly diluted [114]. Nevertheless, mesocarp (flesh) tannins represent as much as skin ones. PAs in the pericarp are mainly based on (–)-epicatechin, (–)-epigallocatechin, (–)-epicatechin-3-*O*-gallate and minor amounts of (+)-catechin, with a polymerization degree around 30 and galloylation rates below 5 % [115]. The regulatory mechanisms of PA accumulation and galloylation are not fully understood, but it is known that biosynthesis occurs exclusively in green stages of berry development [116]. The two trancription factors *VvMybPA1* and *VvMybPA2* were shown to be PA-specific [33, 117]. These TFs more particularly control *Leucoanthocyanidin reductase* (*LAR*) and *anthocyanidin reductase* (*ANR*) at the two branching points of the flavonoid pathway, leading to catechin and epicatechin likely required for formation of PA polymers, and considered as rate limiting enzymes in PA synthesis [117]. The polymerization mechanism remains elusive although major advances were recently made regarding its intracellular localization [145]. Effects of temperature on PA composition of grapevine pericarp are unclear : one study reported an increase of PAs under higher temperatures in one experimental year at only one developmental stage [118].

The present results suggest that temperature impacts tannin synthesis and galloylation in the young berry. Several genes involved in tannin synthesis and its control maintained their expression throughout early developmental stages (G1 and G2) in the cold but were warm down-regulated in G2, such as *VvMybPA2* (VIT_11s0016g01320), which was induced by cool temperature but only in the young green berry (G1) at day and night. Very recently, a transcript named VvMybPAR, showing high protein similarity to VvMybPA2 was shown to intervene as well in the regulation of PA synthesis in young grapevine fruits [119]. However, modulation of this transcript was not confirmed here. Two shikimate dehydrogenase transcripts (VIT_14s0030g00660, VIT_14s0030g00650; Fig. 8), branching the production of flavan-3-ols and gallate, are cold induced as well. A *LAR* transcript (VIT_01s0011g02960) was also highly induced by cool temperatures in the advanced green berry (G2) at day and night (Fig. 8). A similar modulation of these transcripts was already described in berries exposed to short heat stresses [22].

**Fig. 9:**
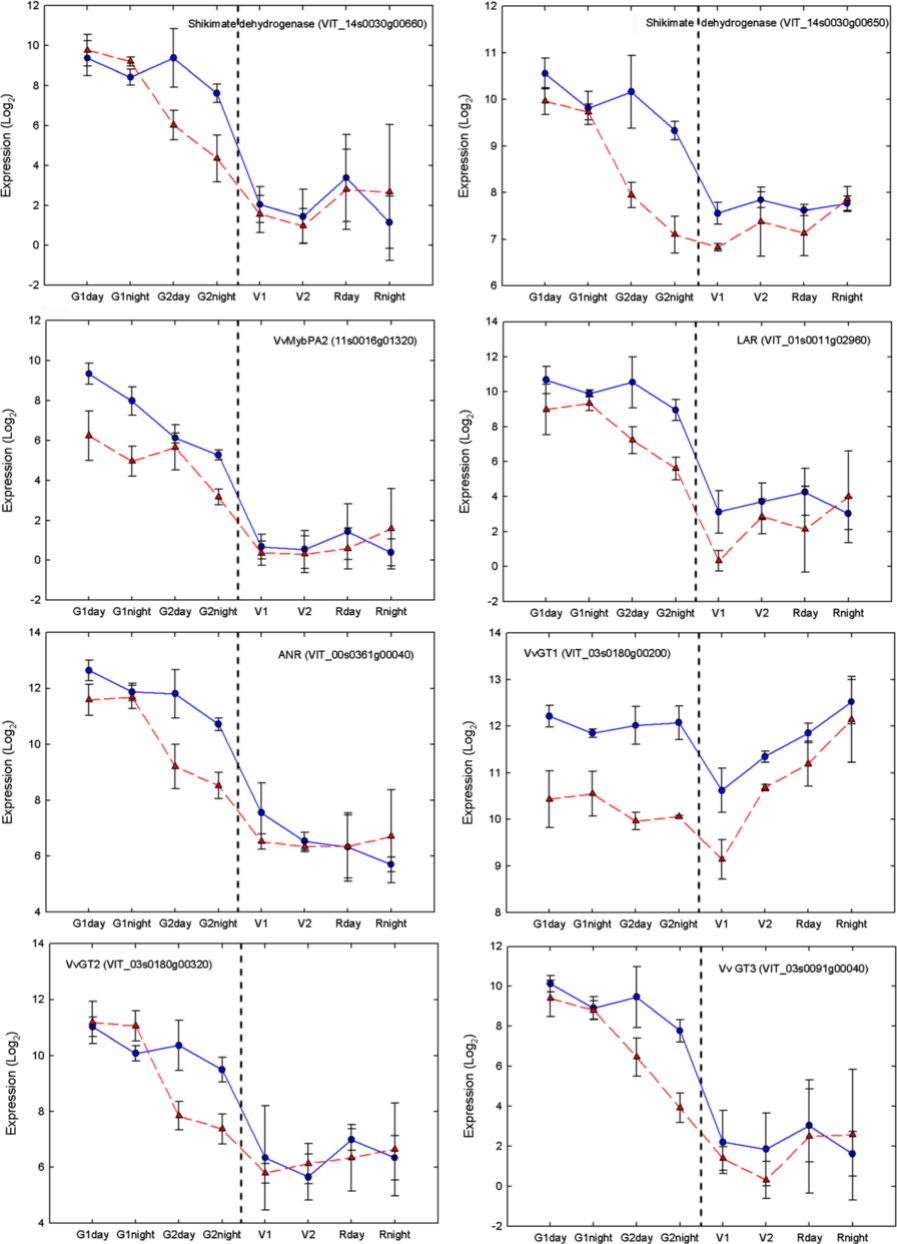
Expression patterns of transcripts in the proanthocyanidin synthesis pathways under different temperatures

Little is known about the mechanisms of tannin polymerization and galloylation. Glycosyltransferases (GTs), which are able to form glucose esters with a wide range of phenolic acids including gallic acid [33], have been suggested to play a role in the galloylation of tannins. Expression of GTs has been shown to parallel PA synthesis and hydroxycinnamic acids esters accumulation before véraison [114]. Three transcripts coding for glycosyltransferases (*VvgGT1, VvgGT2, VvgGT3*), which catalyze the formation of 1-O-acyl-Glc esters of phenolic acids but not of flavanoids and stilbenes were recently suggested to intervene in PA galloylation in berry skin [120]. Transcripts of these GTs (VIT_03s0180g00200, VIT_03s0180g00320, VIT_03s0091g00040; Fig. 9) were actually up-regulated by cold temperature in all developmental stages in the present study, which would indicate increased galloylation under cool temperatures.

The present results suggest that tannin synthesis and galloylation is either impaired by high temperature during the first phase of berry growth or that cool day temperature stimulate tannin synthesis.

### Anthocyanins

As part of the flavonoid group, anthocyanins are plant pigments responsible for red, blue and purple color of plant tissues. Accumulation in grapevine starts with the onset of ripening and is mainly controlled by the transcription factor *VvMybA1* [121, 122]. A key enzyme during the last steps of Anthocyanin biosynthesis in grapes is the UDP-glucose: anthocyanidin: flavonoid glycosyltransferases (*UFGT*), which glycosylate anthocyanin and thereby increases their hydrophilicity and stability [123]. Its expression was only detected in berries after véraison whereas most of the other upstream genes were constitutively expressed in different organs and tissues at diverse levels [124, 125]. Further enzymes involved in anthocyanin accumulation are a O-methyltransferase (OMT, also known as *anthocyanin O-methyltransferase AOMT*), that mediates the methylation of the hydroxyl groups at the C3′ positions or both at the C3′ and C5′ positions on the B rings of the anthocyanins, and GSTs required for transferring anthocyanins into vacuoles [126, 127]. Two multidrug and toxic compound extrusion transporters named VvanthoMATEs were also identified as anthocyanin transporter in grapes, specifically expressed in fruit, concomitantly with the accumulation of anthocyanins, [128].

In plants, anthocyanin accumulation is often enhanced by abiotic stress such as water deficit [106, 110, 111]. In grapevine berries high temperatures during ripening have been found to decrease anthocyanin concentration [129–131], with high night temperature being the most detrimental [132].

Genes involved in anthocyanin biosynthesis did show unambiguous repression by high temperature in previous field studies contrary to detached fruits in vitro [108]. In field experiments, *VvMYBA1* was repressed by heat [133], but this was not confirmed in fruiting cuttings despite the repression by temperature of *anthoMATE*s [70]. In short heat stress studies, most of the genes that correlated with anthocyanin biosynthesis were found heat repressed only on a precisely pre-selected véraison berry stage (*VvMybA1; GST; AOMT1* and *VvanthoMATE3*) [22]. In the present study, a significant modulation of anthocyanin genes could be observed for *VvMybA1* related sequences (VIT_02s0033g00410 and GSVIVT00009898001) repressed by heat at V2. *AnthoMATE3* (VIT_16s0050g00900) and AnthoMATE1 (VIT_16s0050g00930; Additional file 11: Figure S10) were concomitantly repressed in V1. The same expression pattern was observed in temperature studies on fruiting cuttings [23] as well as in short heat stress but only for *anthoMATE 1* in early véraison stage. Statistically, no modulation of *AOMT, UFGT* and *GST* could be evidenced, however a down-regulation tendency can be observed by high temperature (30/25 °C, Day/Night) (Additional file 11: Figure S10A). Furthermore, the expression of those transcripts suggests a delay in the onset of anthocyanin accumulation during the transition from V1 to V2, as observed when plotting log_2_ changes from V1 to V2 in cold condition versus hot condition (Additional file 11: Figure S10B).

Our expression results confirm the impairment and as well the delay of anthocyanin related transcripts. In addition, these data highlight the weight of cluster heterogeneity when ripening starts, since differences in expression could only be observed in V1 and V2, that is to say before the onset of anthocyanin accumulation in berries skins. None of the described transcripts was modulated in R stage. This suggests that a single fruit approach including anthocyanin characterization would be also needed at R stage.

## Conclusions

In the present study, whole grapevine plants were exposed to different temperature gradients for one month periods at least, under fully controlled environment. The consequences on berry development and the evolution of the main osmoticums were described, and related to changes in gene expression observed on day and night samples at five key stages. A total of 674 mio reads were sequenced leading in a total of 10 787 transcripts that were differentially expressed due to temperature. High temperatures induced an acceleration of early berry development and activated several candidate genes reported in previous studies. However, a two-fold change in base 10 growing degree day did not markedly affect the typicall 1.5 month delay between flowering and the onset of ripening. Sample reconstitution through single berry biochemical analysis was performed to mitigate intra-cluster heterogeneity at the onset of ripening and this original procedure considerably improved the accuracy of transcriptomic patterns. Malic acid respiration was favored by heat and genes involved in its membrane transport displayed a marked regulation by both development and temperature. For the first time a decorrelation of malic acid respiration and sugar accumulation was observed in the grapevine fruit at the beginning of ripening. The whole set of data suggests photoassimilate availability plays a key role in the adaptation of the fruit development to environmental changes. A role of consideration of whole plant carbon status and allocation seems to be crucial in further abiotic stress studies.

Secondary metabolism was found repressed by elevated temperature at the transcriptomic level, with a possible impact on the galloylation of proanthocyanidin. In the present study, the regulation of heat shock related genes in the fruit could be precisely deciphered, evidencing their dependence on photo vs. nyctiperiod, as well as, for some of them, their different responses to long-term temperature adaptation, when compared to short heat stress.

## Methods

### Plant material

Dwarf, rapid and continual flowering vines (DRCF, line ML1) [22, 24–27] (Fig. 1) were grown under standard greenhouse conditions until continuous floraison was established on the new phytomers emerging on the proleptic axe (consecutive phytomers are separated by 1.5–3 days, depending on temperature).

### RNAseq experiments

For “green treatments”, all reproductive organs after flowering were removed, before allowing new green berries to develop during a further 28 days period in climatic chambers. Similarly, for “ripening treatments”, all clusters with post-véraison berries were removed before starting the 28 days treatments, during which new berries started to ripen. Applied temperatures were (20/15; 30/15 °C; day/night) for green and (25/15; 30/25 °C) for ripening berries, maintaining all other environmental parameters constant (VPD 1kPa; PAR: 400; photoperiod: 14 h). 10 plants were grown for each nycthemeral temperature regime, among which half of them were sampled at day, and the remaining ones were sampled at night, at the end of experiment.

For “Green Treatments” 30 berries per cluster were sampled, seeds were rapidly removed and berries were immediately frozen in liquid nitrogen. After crushing under N_2_, 100 mg aliquots were used for organic acid and sugar analysis. Three replicates of two stages at day and night of green berry development were chosen according to their sugar and organic acid content for RNA extraction, cDNA library construction and subsequent next generation sequencing.

For “ripening treatments”, the same sampling protocol was applied except for the véraison stages. Berries of all clusters where some berries had just started to soften (as checked by hand) were wrapped individually in an aluminium foil before freezing. These berries were individually crushed, seeds were removed and 50 mg aliquots were used for organic acid and sugar analysis. Precising the developmental stage of each individual berry (Fig. 3) served to form several homogeneous batches for RNAseq analysis. For clusters where all berries have turned red, no single berry selection was performed but 30 berries per cluster were sampled, before seeds removal, immediate freezing, crushing and solute analysis.

### Blocking malate breakdown by cold temperature

Plants were grown in greenhouses until reaching 55 expanded leaves. All post-veraison clusters were removed. 8 plants were then cultivated at 22–13 °C, and 8 plants at 30–20 °C, during 58 and 29 days respectively, which allowed 20 new phytomers to develop in both temperature conditions. All clusters from all plants were analyzed separately, from anthesis, to late ripening.

Plant growth was assessed by weekly countings of leaves. Leaf emergence rate (LER) of ca 24 °C.day.leaf^−1^ was inferred from the linear regression between leaf number and cumulated thermal time, with a base temperature of 10 °C [95]. The age of each cluster was then obtained from its position using the following relation:

Cluster age (Julian days after anthesis) = [(1 ÷ LER) ÷ TTday)] x phytomer where phytomer is the position of the cluster counted from the last flowers at anthesis.

### Organic acid and sugar analysis

50–100 mg of N_2_-ground powder was diluted (5 fold) and frozen at −20 °C. Samples were heated (60 °C for 30 min), subsequently homogenized and diluted in 4.375 μM acetate as internal standard. 0.18 g of Sigma Amberlite IR-120 Plus (sodium form) (Sigma-Aldrich, St. Louis, MO, USA) was added to 1 mL of sample (prevention of potassium bitartrate precipitation) before agitation in a rotary shaker for at least 10 h. Samples were centrifuged (15 493 g for 10 min) and the supernatant was transferred into high-performance liquid chromatography (HPLC) vials before injection onto an Aminex HPX87H column (Bio-Rad, Marnes-la-Coquette, France) eluted under isocratic conditions (0.5 mL/min, 60 °C, 5 mM H_2_SO_4_) [134]. UV absorbance (210 nm) and refractive index were measured with a Waters 2487 dual absorbance detector (Waters Corporation, Milford, MA, USA) in series with a Kontron 475 detector (Kontron Instruments, Rossdorf, Germany) and the concentration of hexoses and organic acids were calculated according to [135].

### RNA extraction

RNA extraction was performed as in Rienth et al*.*, [136]: Five ml extraction buffer (6 M guanidine-hydrochloride, 0.15 M tri-sodium-citrate, 20 mM EDTA and 1.5 % CTAB) were added to 1 g of powder followed by immediate agitation. Cell debris was removed by centrifugation, after chloroform washing, one volume of isopropanol was added to precipitate RNA. Samples were kept at – 20 °C for at least two hours. Precipitated RNA was separated by centrifugation and cleaned with 75 % ethanol. The pellet was re-suspended in RLC buffer from the Quiagen RNAeasy® Kit and an additional chloroform-cleaning step was undergone. The succeeding washing steps and the DNAse treatment were performed as described in the kit. Absorbances at 260 and 280 nm were measured with a NanoDrop 2000c Spectrophotometer Thermo Scientific®. The integrity of RNA was determined using a 2100 Bioanalyzer (Agilent Technolgies®).

### RNA sequencing

cDNA synthesis of fragmented RNA was performed according to the Illumina TruSeq RNA sample preparation kit (low-throughput protocol) (Illumina, Inc., San Diego, CA, USA). cDNA was synthesized from enriched and fragmented RNA using reverse transcriptase (Super-Script II, Illumina) and random primers. This was followed by a conversion into double-stranded DNA using the reagents supplied in the kit, and the resulting DNA was used for library preparation. Blunt ends of cDNAs were modified with an addition of a 3-adenine and modified ends were then used to ligate an indexed adapter. This allowed samples to be distinguished from each other during flow-cell hybridization and sequencing. The subsequent adapter-modified DNA fragments were then enriched using standard PCR.

Cluster generation of cDNA libraries and hybridizations onto the flow-cell was performed with the Illumina cluster generation kit (Illumina). Paired-end sequencing was realized on a HiSeq 2000 Illumina sequencer using SBS (Sequence By Synthesis) technology (Illumina). Image analysis was carried out with Illumina HiSeq Control Software (Illumina), which identifies cluster position, intensity and background noise. Intensity was transformed into nucleotide bases by RTA software from Illumina.

Obtained reads were pre-processed with cutadapt [137] using the TruSeq index sequence corresponding to each sample. End of reads with low quality scores (parameter -q 20) were trimmed with cutadapt and reads with a minimum length of 35 bp were kept. Subsequently reads were filtered according to their mean quality score, keeping those higher than 30. Orphan reads (i.e. those for which the mate was discarded in the previous steps) were then separated using a homemade script. RNA data is available in the SRA system of the NCBI under the project SRP059734 (http://www.ncbi.nlm.nih.gov/sra/).

Cleaned reads (paired and orphan) from each library were mapped on the reference from [138] with BWA [139] allowing 3 errors (-n 3 in the aln step). PCR and optical duplicates were removed with the picard tools (http://broadinstitute.github.io/picard/). For each reference sequence and each sample, raw read counts were obtained using the SAMtools [140].

Gene counts were computed by summing counts of different transcripts of the same gene. Differentially expressed (DE) genes were identified using the R package DESeq [141]. FDR of the Benjamini-Hochberg multiple tests of 1 % (*P* < 0.01) based on read counts and a minimum fold-change of two (log_2_ fold change < -1; > + 1) in at least one pairwise comparison. Pairwise comparison was performed between low and high temperatures in all condition separately, day and night. To follow developmentally regulated transcripts an additional pairwise comparison was performed between two consecutive stages at either cool or hot condition for day and night separately.

All temperature modulated transcripts were extracted and clustered with Multiple Experiment Viewer® version 4.6.2, by hierarchical clustering using Pearson’s correlation distance calculated on normalized and mean centered count data. Principal component analysis (PCA) was performed with R version 2.14.0 (R Development Core Team) on average base means of each triplicate. To identify transcripts that were differentially modulated by temperature in relation to day or night-time a maSigPro two-class time series comparison (polynomial degree 2) [43] was conducted using daily average temperatures of treatments as quantitative variable and time as qualitative parameter. *P* values of 0.05 were chosen as significance levels for gene selection after Benjamini-Hochberg correction and for model variable in maSigPro.

A first global analysis of functional categories was done on all heat modulated transcripts in all stages and conditions, using the pageman software [142] implemented in Mapman [143], the mapping file was derived from http://www.gomapman.org/.

Subsequently all specifically modulated transcripts were extracted and analyzed using the FatiGO analysis tool [144], to compare the genes list with non-redundant transcripts from the grapevine genome using a Fisher’s exact test. Significant enrichment was considered in case of *p* value < 0.01 and illustrated as fold change.

## Abbreviations

ABA, abcissic Acid; AHA, Arabidospsis H^+^ -ATPase; ALMT, aluminium-activated malate transporter; CHS, Chalcone Synthase; DAA, Day After Anthesis; DEG, Differentially Expressed Gene; DRCF, Dwarf Rapid Cycling and Flowering; Exp, Expansin; G (1 or 2), green stage (1 or 2); GT, Glucosyl Transferase; HSF, Heat Stress transcription Factor; HSG, Heat Stress Gene; HSP, Heat Stress Protein; LAR, Leuco-Anthocyanidin Reductase; MA, Malic Acid; MIP, Membrane Intrinsic Protein; N, Night; PA, Proanthocyanidin; PAL, Phenylalanin Ammonia Lyase; PAR, Photosynthetically Active Radiation; PCA, PC, Principal Componant Analysis, Principal Component; PIP, Plasma membrane Intrinsic Protein; R, Ripe stage; TA, Tartric Acid; V (1or 2), Véraison (1 or 2); VPD, Vapor Pressure Deficit

## Additional files

Additional file 1: Figure S1. (A) Malate, (B) Tartrate, (C) Sugar concentrations and (D) Berry Weight of microvine clusters following prolonged 30–20 °C or 22–12 °C growth periods. Each point represents one cluster (~30 berries). At the end of each treatment, all clusters were harvested simultaneously on 5 independent plants. The induction of ripening is marked by the simultaneous inductions of malate breakdown and massive hexose storage in berries at 30–20 °C, as typically observed in vineyard conditions. The onset of malate breakdown is delayed to 0.4–0.5 M hexoses following a 3 months growth period at 22–12 °C (DAF, Days After Flowering). (PDF 175 kb) Additional file 2: Table S1. Differentially expressed transcripts (lfc > 1, padj < 0.05) between the two temperature gradients at all stages and times. (XLSX 2237 kb) Additional file 3: Figure S2. Hierarchical clustering of normalized counts of all differentially expressed transcripts upon high temperatures including all replicates. (TIF 2433 kb) Additional file 4: Figure S3. Principal component analysis on all averaged normalized counts of all conditions, stages and time points. (TIF 576 kb) Additional file 5: Figure S4. Expression of day/night inversed transcripts upon temperature in R stage. (BMP 515 kb) Additional file 6: Figure S5. A-C: Functional categories of heat stress modulated transcripts in all stages and time points (blue: heat induced, red: heat: heat repressed). (PDF 1374 kb) Additional file 7: Figure S6. Functional categories of stage and time specifically temperature modulated transcripts. (JPG 46 kb) Additional file 8: Table S2. Differentially expressed transcripts with clusters identified by time series analysis. (XLSX 589 kb) Additional file 9: Figure S8. Expression profiles of transcripts related to berry weight. (TIF 577 kb) Additional file 10: Figure S9. Phylogeny of *Vitis* Aluminium Activated Malate Transporters: *Vitis vinifera* was lacking in previous detailed clade analysis [88] because obsolete gene models were considered. *Vitis* ALMTs from Fig. 1 in [86], MDP25214 and MDP244249 genes close to apple Ma1 locus [87] are included here, using similar sequence blocks than in [88]. The latest ALMT sequences predicted in *Vitis* were retrieved by BLAST from RefSeq NCBI Annotation release 101 (ftp://ftp.ncbi.nih.gov/genomes/Vitis_vinifera/protein/) and from CRIBI V2 (http://genomes.cribi.unipd.it/DATA/). Two conflicts emerged between databanks: (1) XP_010647826.1 (or XP_002278994.2), and XP_002278978.1 were concatenated in VIT 201s0011g03290. Alignment of our pair end sequencing data (not shown) clearly argue in favor of Refseq predictions and confirms the existence of separated VviALMT5&6 peptides, as in [86]. (2) XP_003632294 (435 aa) appears more pertinent than VIT_206s0080g00200.1 (283 aa), regarding VviALMT4. Genes were renamed according to the directives of the International Grape Genome Project consortium, keeping the same numbers than in [86] (ie VviALMT6 replaces VVALMT6). (PDF 57 kb) Additional file 11: Figure S10. A) Expression profiles of transcripts involved in Anthocyanin synthesis: B) Véraison stage comparison of Anthocyanin transcripts: Log2 V1_hot/V2_hot vs. V1_cold/V2 cold. (TIF 380 kb) Additional file 12: Figure S7. HCL clustering of DEGs identified by time series analysis. (TIF 126 kb)
