# Supplementary figures and images for: Temperature desynchronizes sugar and organic acid metabolism in ripening grapevine fruits and remodels their transcriptome
Source: BMC Plant Biol. 2016 Jul 20;16:164. doi: 10.1186/s12870-016-0850-0 (PMC4955140; doi:10.1186/s12870-016-0850-0)

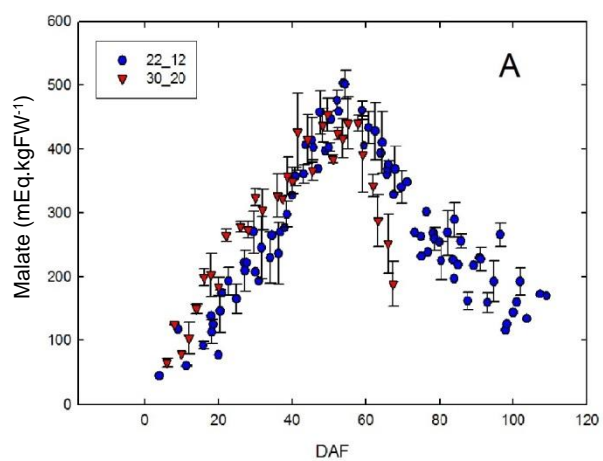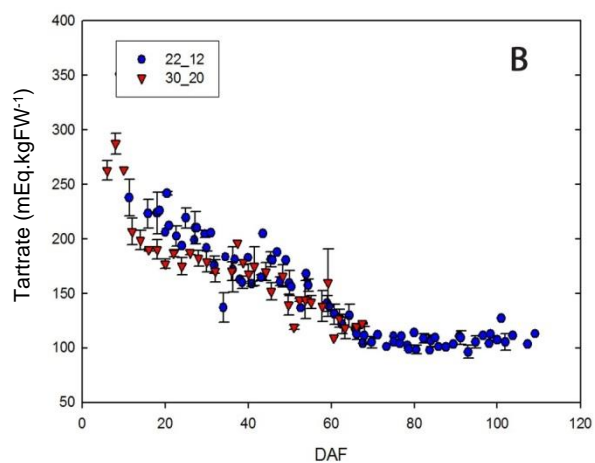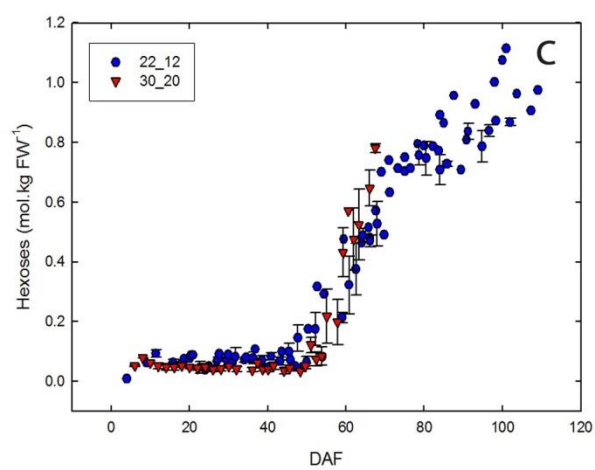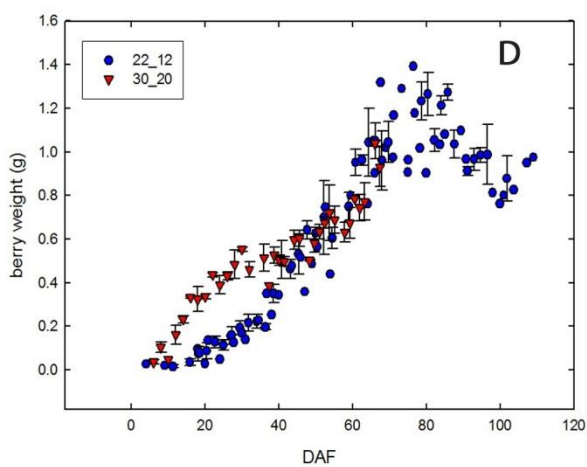

Supplement: Additional file 1: Figure S1. — (A) Malate, (B) Tartrate, (C) Sugar concentrations and (D) Berry Weight of microvine clusters following prolonged 30–20 °C or 22–12 °C growth periods. Each point represents one cluster (~30 berries). At the end of each treatment, all clusters were harvested simultaneously on 5 independent plants. The induction of ripening is marked by the simultaneous inductions of malate breakdown and massive hexose storage in berries at 30–20 °C, as typically observed in vineyard conditions. The onset of malate breakdown is delayed to 0.4–0.5 M hexoses following a 3 months growth period at 22–12 °C (DAF, Days After Flowering). (PDF 175 kb) [file 12870_2016_850_MOESM1_ESM.pdf]

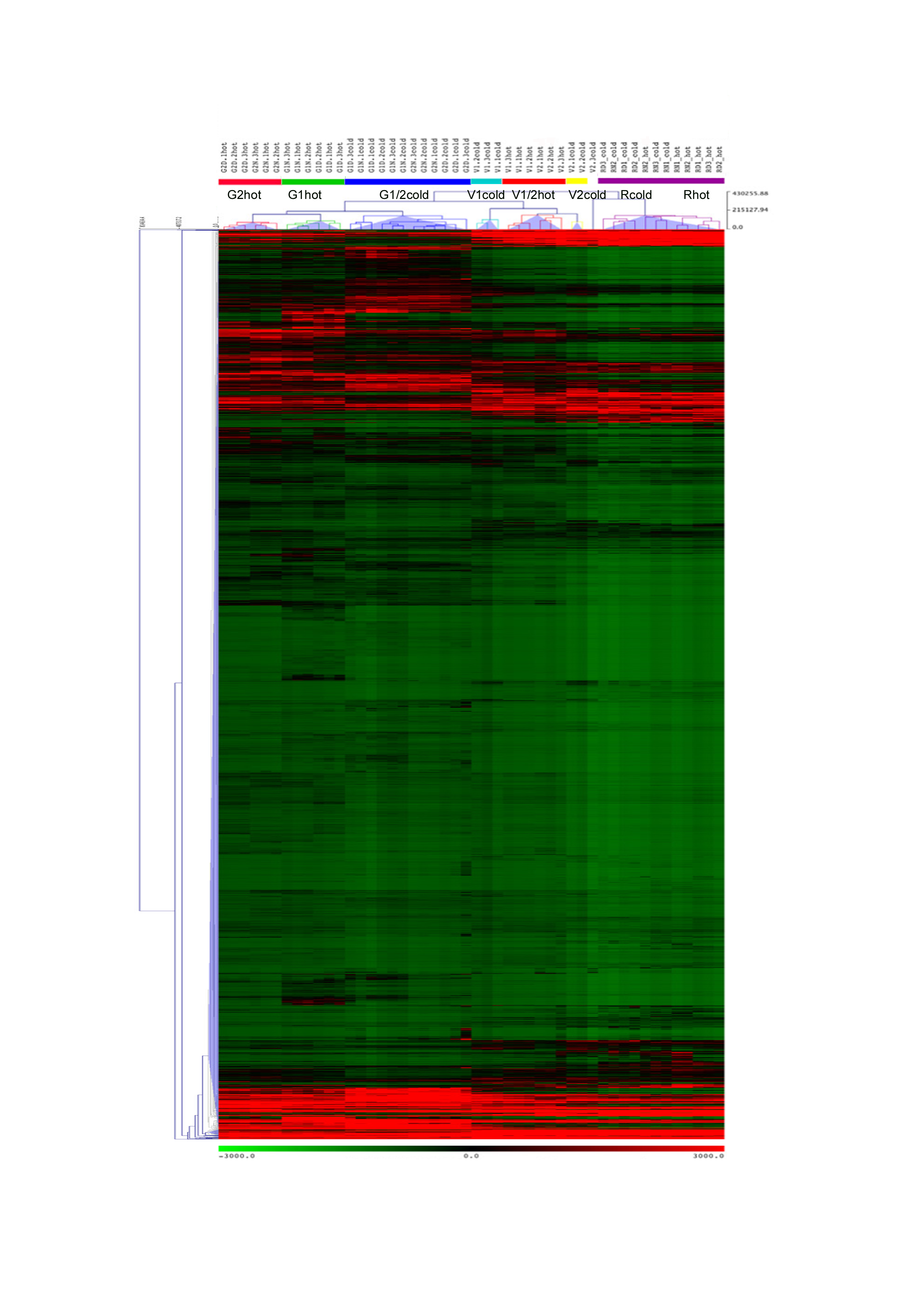

Supplement: Additional file 3: Figure S2. — Hierarchical clustering of normalized counts of all differentially expressed transcripts upon high temperatures including all replicates. (TIF 2433 kb) [file 12870_2016_850_MOESM3_ESM.tif]

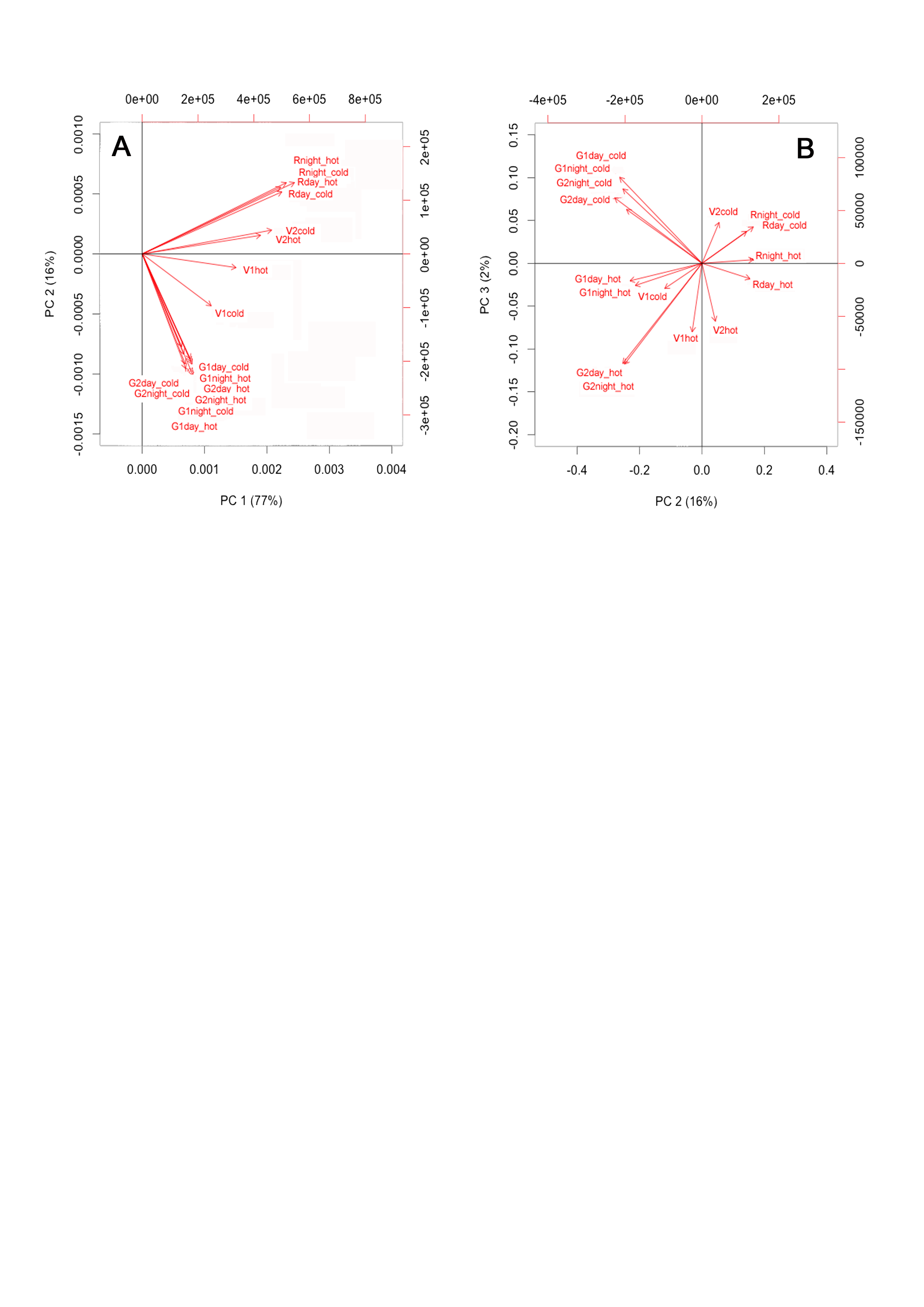

Supplement: Additional file 4: Figure S3. — Principal component analysis on all averaged normalized counts of all conditions, stages and time points. (TIF 576 kb) [file 12870_2016_850_MOESM4_ESM.tif]

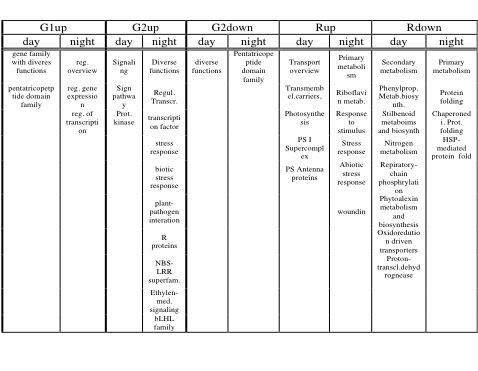

Supplement: Additional file 7: Figure S6. — Functional categories of stage and time specifically temperature modulated transcripts. (JPG 46 kb) [file 12870_2016_850_MOESM7_ESM.jpg]

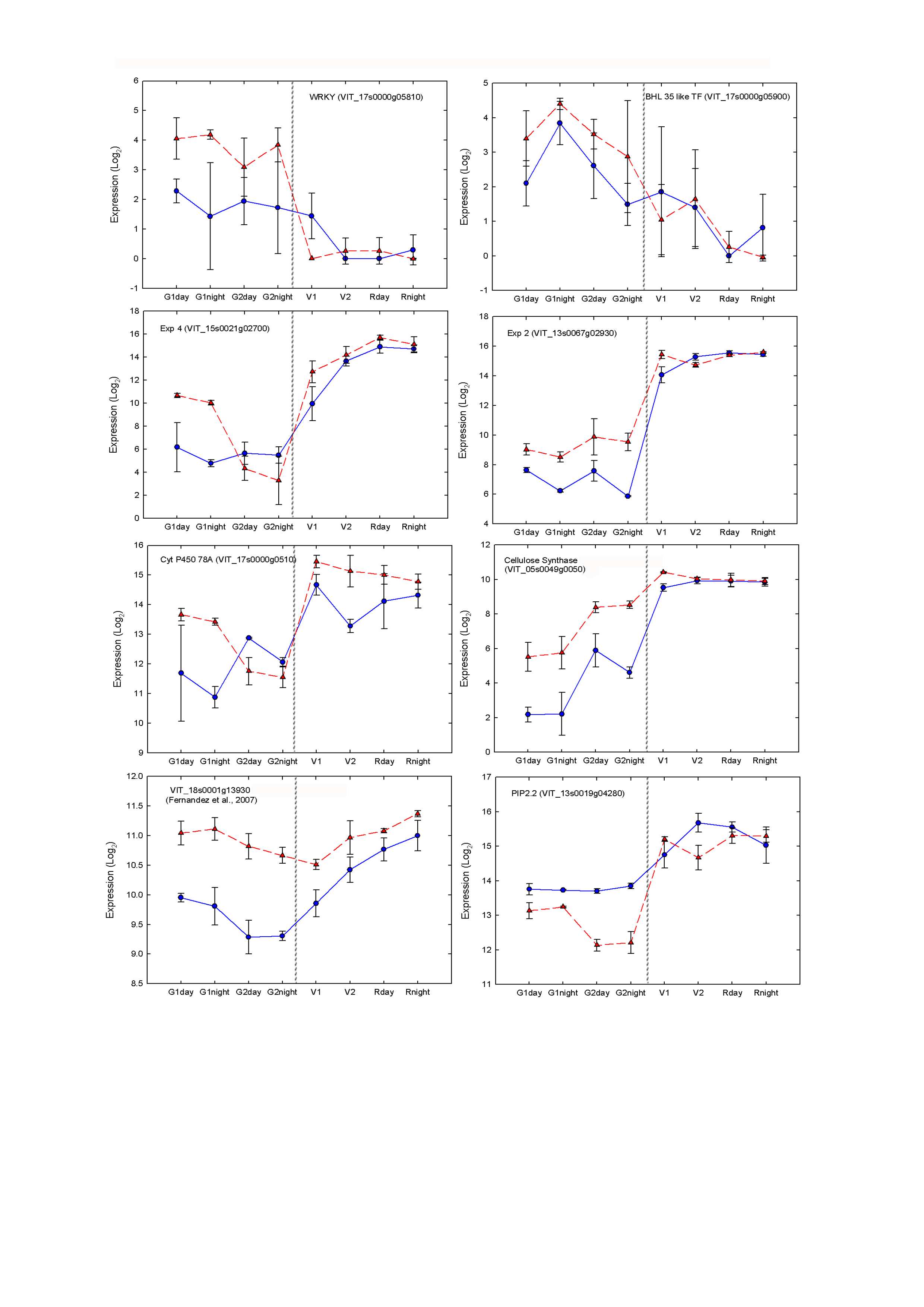

Supplement: Additional file 9: Figure S8. — Expression profiles of transcripts related to berry weight. (TIF 577 kb) [file 12870_2016_850_MOESM9_ESM.tif]

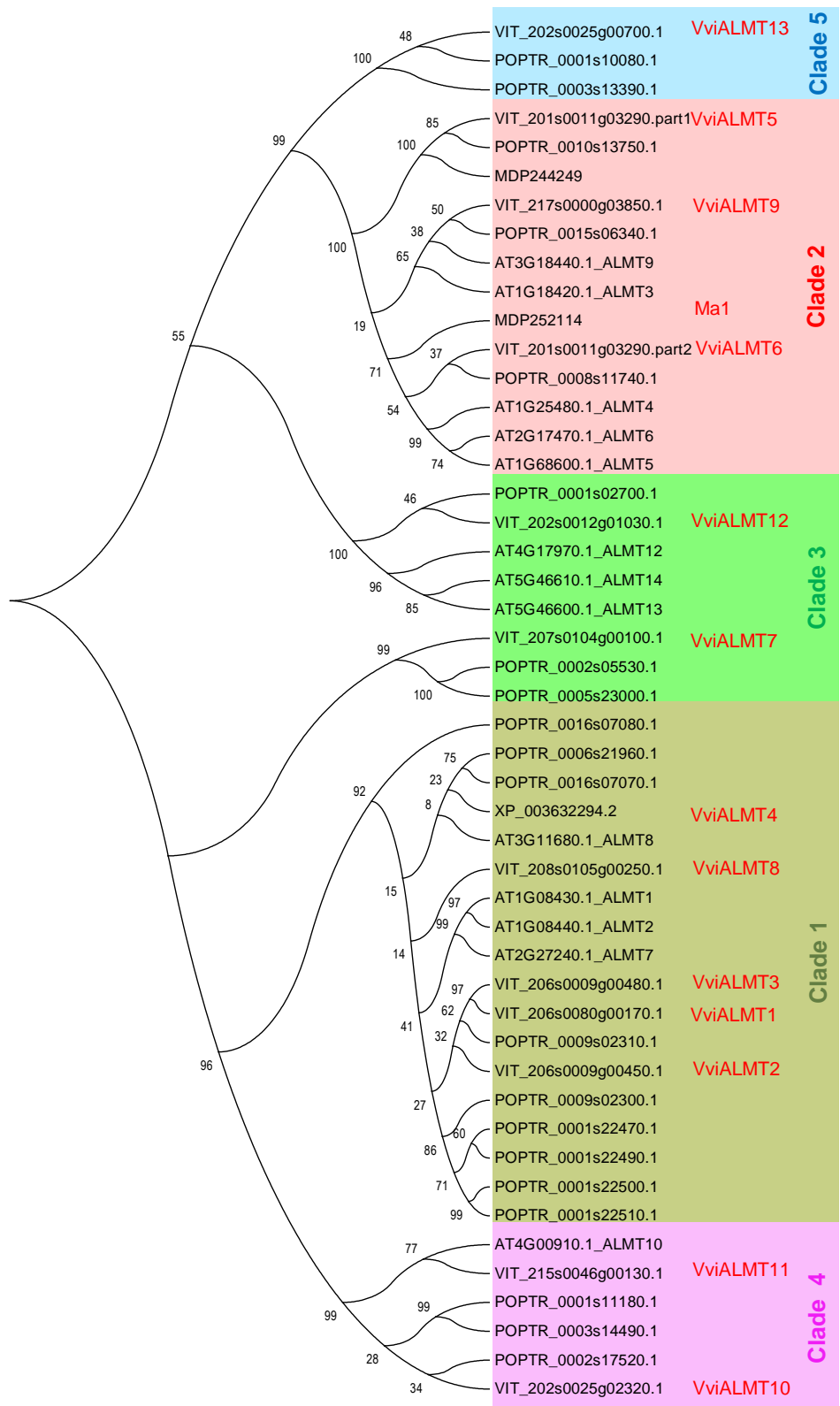

Supplement: Additional file 10: Figure S9. — Phylogeny of Vitis Aluminium Activated Malate Transporters: Vitis vinifera was lacking in previous detailed clade analysis [88] because obsolete gene models were considered. Vitis ALMTs from Fig. 1 in [86], MDP25214 and MDP244249 genes close to apple Ma1 locus [87] are included here, using similar sequence blocks than in [88]. The latest ALMT sequences predicted in Vitis were retrieved by BLAST from RefSeq NCBI Annotation release 101 (ftp://ftp.ncbi.nih.gov/genomes/Vitis_vinifera/protein/) and from CRIBI V2 (http://genomes.cribi.unipd.it/DATA/). Two conflicts emerged between databanks: (1) XP_010647826.1 (or XP_002278994.2), and XP_002278978.1 were concatenated in VIT 201s0011g03290. Alignment of our pair end sequencing data (not shown) clearly argue in favor of Refseq predictions and confirms the existence of separated VviALMT5&6 peptides, as in [86]. (2) XP_003632294 (435 aa) appears more pertinent than VIT_206s0080g00200.1 (283 aa), regarding VviALMT4. Genes were renamed according to the directives of the International Grape Genome Project consortium, keeping the same numbers than in [86] (ie VviALMT6 replaces VVALMT6). (PDF 57 kb) [file 12870_2016_850_MOESM10_ESM.pdf]

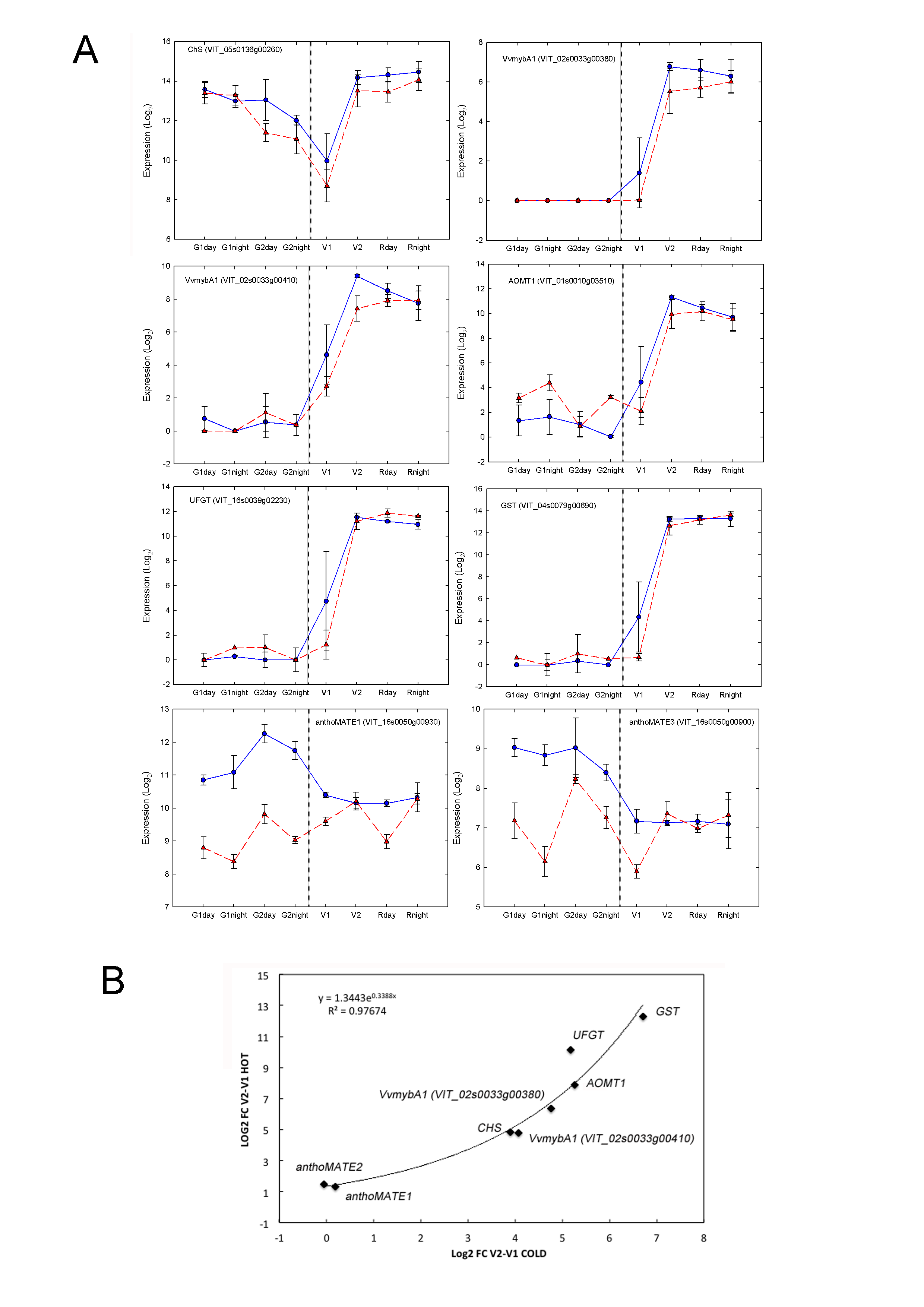

Supplement: Additional file 11: Figure S10. — A) Expression profiles of transcripts involved in Anthocyanin synthesis: B) Véraison stage comparison of Anthocyanin transcripts: Log2 V1_hot/V2_hot vs. V1_cold/V2 cold. (TIF 380 kb) [file 12870_2016_850_MOESM11_ESM.tif]

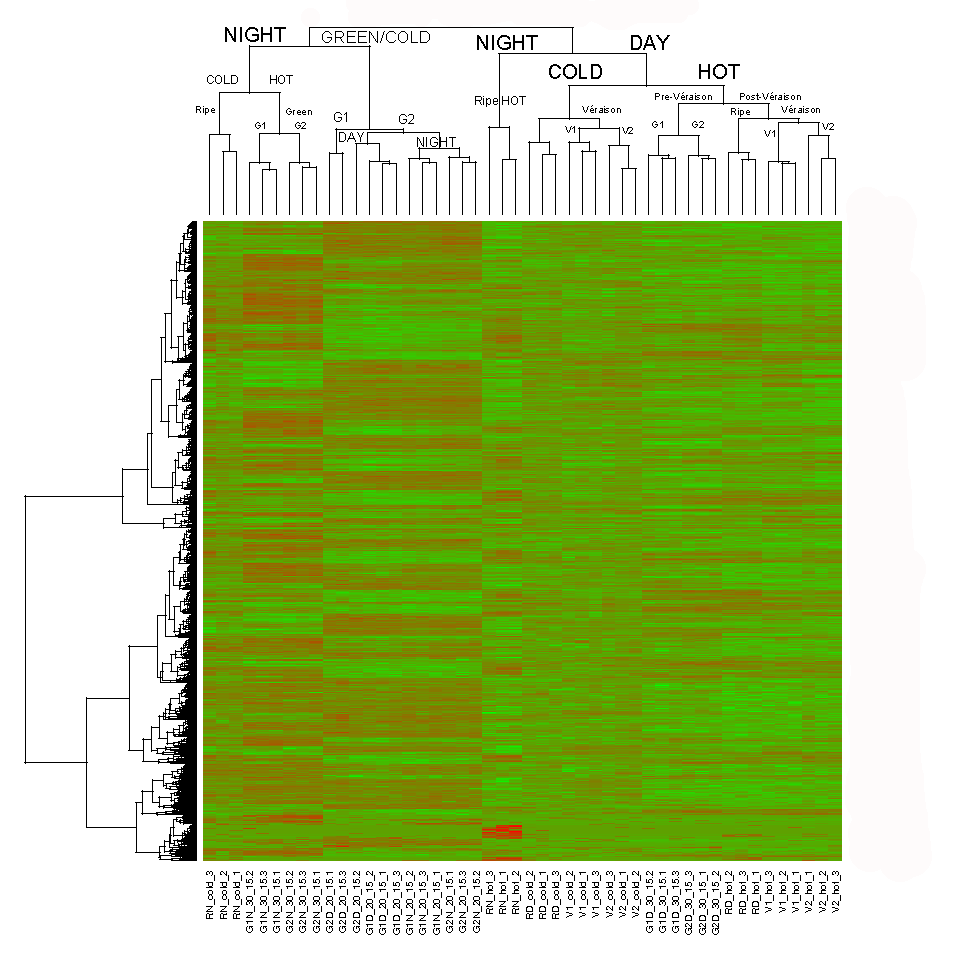

Supplement: Additional file 12: Figure S7. — HCL clustering of DEGs identified by time series analysis. (TIF 126 kb) [file 12870_2016_850_MOESM12_ESM.tif]
