# Supplementary material for: Temperature desynchronizes sugar and organic acid metabolism in ripening grapevine fruits and remodels their transcriptome
Source: BMC Plant Biol. 2016 Jul 20;16:164. doi: 10.1186/s12870-016-0850-0 (PMC4955140; doi:10.1186/s12870-016-0850-0)
Supplement: Additional file 6: Figure S5. — A-C: Functional categories of heat stress modulated transcripts in all stages and time points (blue: heat induced, red: heat: heat repressed). (PDF 1374 kb) [file 12870_2016_850_MOESM6_ESM.pdf]

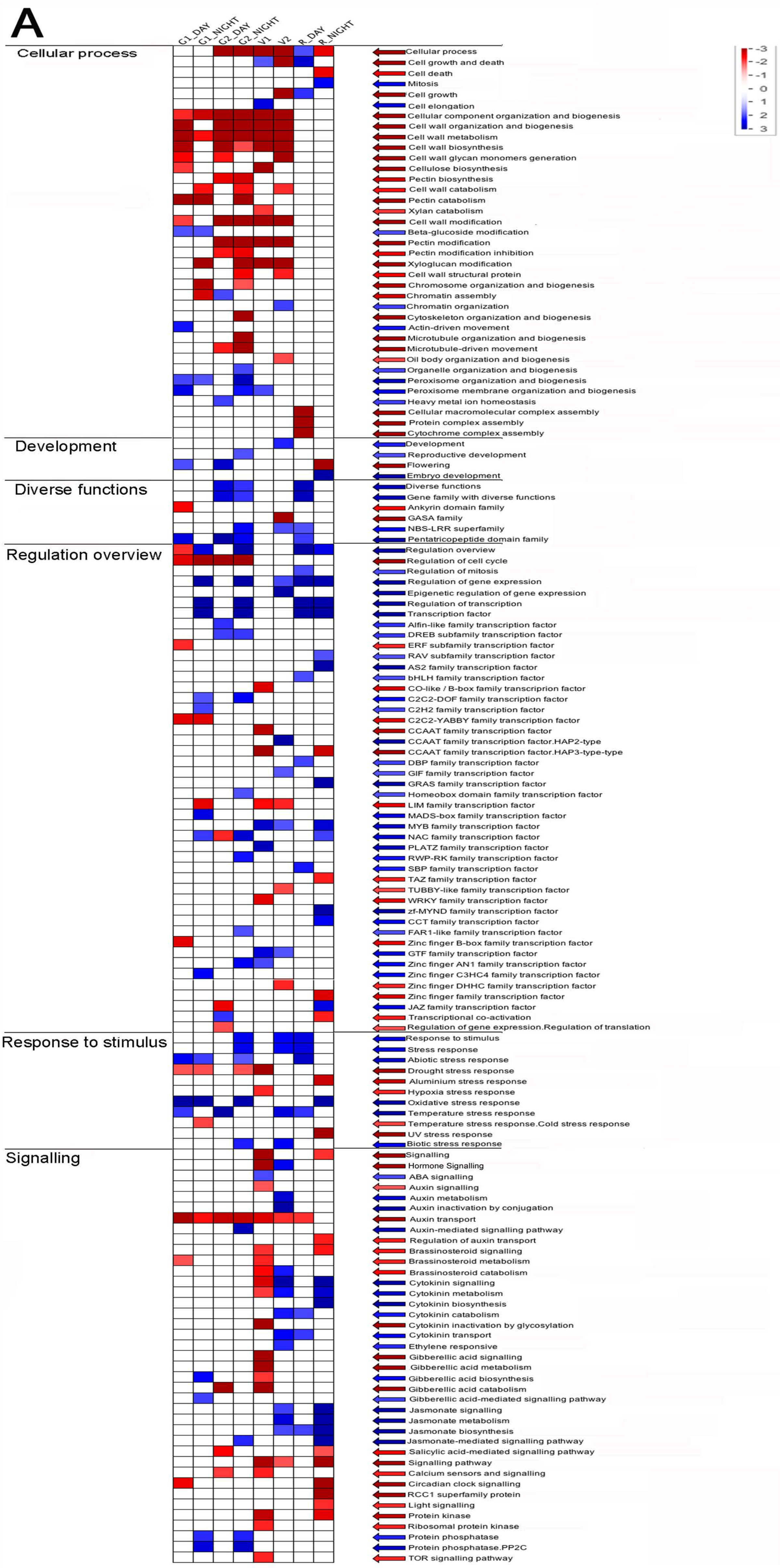
A
G1_DAY
G1_NIGHT
G2_DAY
G2_NIGHT
V1
V2
R_DAY
R_NIGHT
-3
-2
-1
0
1
2
3
Cellular process
Development
Diverse functions
Regulation overview
Response to stimulus
Signalling
Cellular process
Cell growth and death
Cell death
Mitosis
Cell growth
Cell elongation
Cellular component organization and biogenesis
Cell wall organization and biogenesis
Cell wall metabolism
Cell wall biosynthesis
Cell wall glycan monomers generation
Cellulose biosynthesis
Pectin biosynthesis
Cell wall catabolism
Pectin catabolism
Xylan catabolism
Cell wall modification
Beta-glucoside modification
Pectin modification
Pectin modification inhibition
Xyloglucan modification
Cell wall structural protein
Chromosome organization and biogenesis
Chromatin assembly
Chromatin organization
Cytoskeleton organization and biogenesis
Actin-driven movement
Microtubule organization and biogenesis
Microtubule-driven movement
Oil body organization and biogenesis
Organelle organization and biogenesis
Peroxisome organization and biogenesis
Peroxisome membrane organization and biogenesis
Heavy metal ion homeostasis
Cellular macromolecular complex assembly
Protein complex assembly
Cytochrome complex assembly
Development
Reproductive development
Flowering
Embryo development
Diverse functions
Gene family with diverse functions
Ankyrin domain family
GASA family
NBS-LRR superfamily
Pentatricopeptide domain family
Regulation overview
Regulation of cell cycle
Regulation of mitosis
Regulation of gene expression
Epigenetic regulation of gene expression
Regulation of transcription
Transcription factor
Alfin-like family transcription factor
DREB subfamily transcription factor
ERF subfamily transcription factor
RAV subfamily transcription factor
AS2 family transcription factor
bHLH family transcription factor
CO-like / B-box family transcriprion factor
C2C2-DOF family transcription factor
C2H2 family transcription factor
C2C2-YABBY family transcription factor
CCAAT family transcription factor
CCAAT family transcription factor.HAP2-type
CCAAT family transcription factor.HAP3-type-type
DBP family transcription factor
GIF family transcription factor
GRAS family transcription factor
Homeobox domain family transcription factor
LIM family transcription factor
MADS-box family transcription factor
MYB family transcription factor
NAC family transcription factor
PLATZ family transcription factor
RWP-RK family transcription factor
SBP family transcription factor
TAZ family transcription factor
TUBBY-like family transcription factor
WRKY family transcription factor
zf-MYND family transcription factor
CCT family transcription factor
FAR1-like family transcription factor
Zinc finger B-box family transcription factor
GTF family transcription factor
Zinc finger AN1 family transcription factor
Zinc finger C3HC4 family transcription factor
Zinc finger DHHC family transcription factor
Zinc finger family transcription factor
JAZ family transcription factor
Transcriptional co-activation
Regulation of gene expression.Regulation of translation
Response to stimulus
Stress response
Abiotic stress response
Drought stress response
Aluminium stress response
Hypoxia stress response
Oxidative stress response
Temperature stress response
Temperature stress response.Cold stress response
UV stress response
Biotic stress response
Signalling
Hormone Signalling
ABA signalling
Auxin signalling
Auxin metabolism
Auxin inactivation by conjugation
Auxin transport
Auxin-mediated signalling pathway
Regulation of auxin transport
Brassinosteroid signalling
Brassinosteroid metabolism
Brassinosteroid catabolism
Cytokinin signalling
Cytokinin metabolism
Cytokinin biosynthesis
Cytokinin catabolism
Cytokinin inactivation by glycosylation
Cytokinin transport
Ethylene responsive
Gibberellic acid signalling
Gibberellic acid metabolism
Gibberellic acid biosynthesis
Gibberellic acid catabolism
Gibberellic acid-mediated signalling pathway
Jasmonate signalling
Jasmonate metabolism
Jasmonate biosynthesis
Jasmonate-mediated signalling pathway
Salicylic acid-mediated signalling pathway
Signalling pathway
Calcium sensors and signalling
Circadian clock signalling
RCC1 superfamily protein
Light signalling
Protein kinase
Ribosomal protein kinase
Protein phosphatase
Protein phosphatase.PP2C
TOR signalling pathway

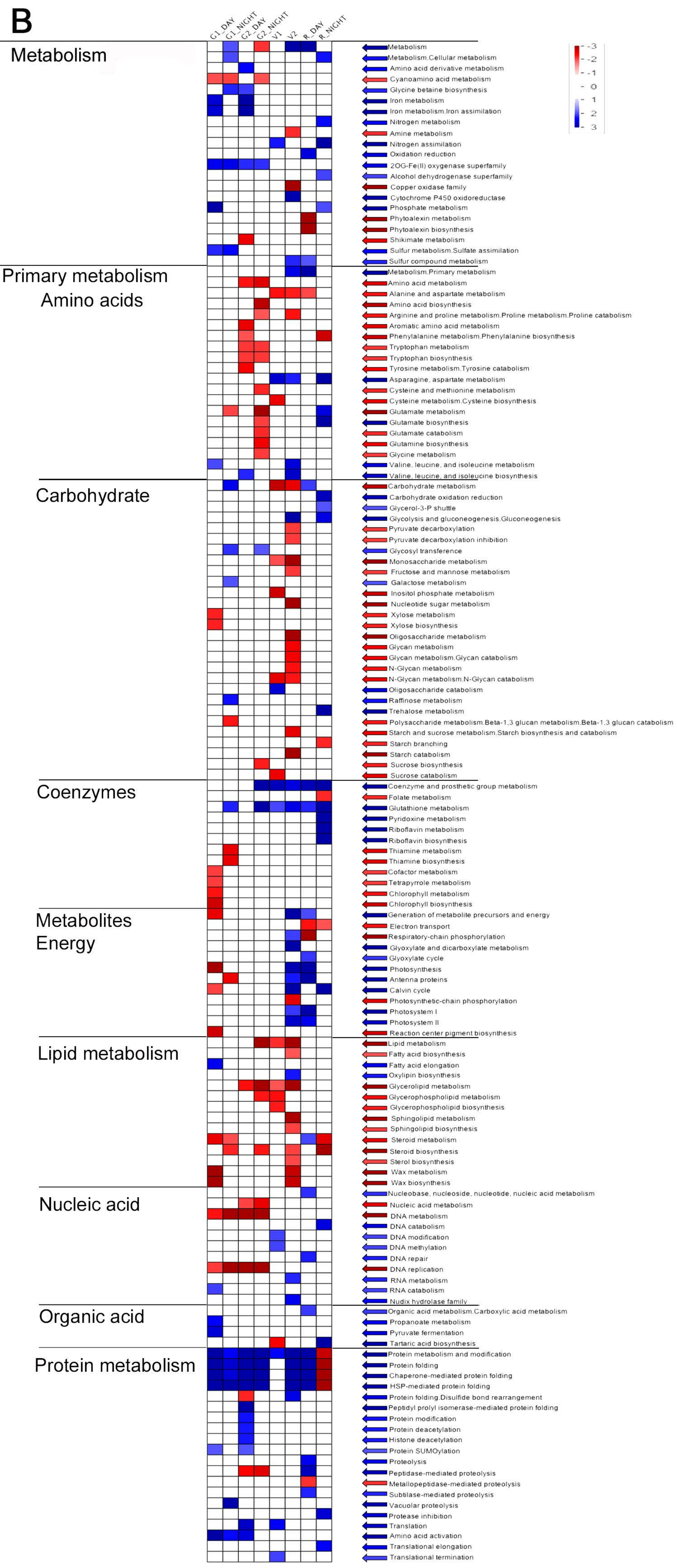
B
G1_DAY
G1_NIGHT
G2_DAY
G2_NIGHT
V1
V2
R_DAY
R_NIGHT
-3
-2
-1
0
1
2
3
Metabolism
Primary metabolism
Amino acids
Carbohydrate
Coenzymes
Metabolites
Energy
Lipid metabolism
Nucleic acid
Organic acid
Protein metabolism
Metabolism
Metabolism.Cellular metabolism
Amino acid derivative metabolism
Cyanoamino acid metabolism
Glycine betaine biosynthesis
Iron metabolism
Iron metabolism.Iron assimilation
Nitrogen metabolism
Amine metabolism
Nitrogen assimilation
Oxidation reduction
2OG-Fe(II) oxygenase superfamily
Alcohol dehydrogenase superfamily
Copper oxidase family
Cytochrome P450 oxidoreductase
Phosphate metabolism
Phytoalexin metabolism
Phytoalexin biosynthesis
Shikimate metabolism
Sulfur metabolism.Sulfate assimilation
Sulfur compound metabolism
Metabolism.Primary metabolism
Amino acid metabolism
Alanine and aspartate metabolism
Amino acid biosynthesis
Arginine and proline metabolism.Proline metabolism.Proline catabolism
Aromatic amino acid metabolism
Phenylalanine metabolism.Phenylalanine biosynthesis
Tryptophan metabolism
Tryptophan biosynthesis
Tyrosine metabolism.Tyrosine catabolism
Asparagine, aspartate metabolism
Cysteine and methionine metabolism
Cysteine metabolism.Cysteine biosynthesis
Glutamate metabolism
Glutamate biosynthesis
Glutamate catabolism
Glutamine biosynthesis
Glycine metabolism
Valine, leucine, and isoleucine metabolism
Valine, leucine, and isoleucine biosynthesis
Carbohydrate metabolism
Carbohydrate oxidation reduction
Glycerol-3-P shuttle
Glycolysis and gluconeogenesis.Gluconeogenesis
Pyruvate decarboxylation
Pyruvate decarboxylation inhibition
Glycosyl transference
Monosaccharide metabolism
Fructose and mannose metabolism
Galactose metabolism
Inositol phosphate metabolism
Nucleotide sugar metabolism
Xylose metabolism
Xylose biosynthesis
Oligosaccharide metabolism
Glycan metabolism
Glycan metabolism.Glycan catabolism
N-Glycan metabolism
N-Glycan metabolism.N-Glycan catabolism
Oligosaccharide catabolism
Raffinose metabolism
Trehalose metabolism
Polysaccharide metabolism.Beta-1,3 glucan metabolism.Beta-1,3 glucan catabolism
Starch and sucrose metabolism.Starch biosynthesis and catabolism
Starch branching
Starch catabolism
Sucrose biosynthesis
Sucrose catabolism
Coenzyme and prosthetic group metabolism
Folate metabolism
Glutathione metabolism
Pyridoxine metabolism
Riboflavin metabolism
Riboflavin biosynthesis
Thiamine metabolism
Thiamine biosynthesis
Cofactor metabolism
Tetrapyrrole metabolism
Chlorophyll metabolism
Chlorophyll biosynthesis
Generation of metabolite precursors and energy
Electron transport
Respiratory-chain phosphorylation
Glyoxylate and dicarboxylate metabolism
Glyoxylate cycle
Photosynthesis
Antenna proteins
Calvin cycle
Photosynthetic-chain phosphorylation
Photosystem I
Photosystem II
Reaction center pigment biosynthesis
Lipid metabolism
Fatty acid biosynthesis
Fatty acid elongation
Oxylipin biosynthesis
Glycerolipid metabolism
Glycerophospholipid metabolism
Glycerophospholipid biosynthesis
Sphingolipid metabolism
Sphingolipid biosynthesis
Steroid metabolism
Steroid biosynthesis
Sterol biosynthesis
Wax metabolism
Wax biosynthesis
Nucleobase, nucleoside, nucleotide, nucleic acid metabolism
Nucleic acid metabolism
DNA metabolism
DNA catabolism
DNA modification
DNA methylation
DNA repair
DNA replication
RNA metabolism
RNA catabolism
Nudix hydrolase family
Organic acid metabolism.Carboxylic acid metabolism
Propanoate metabolism
Pyruvate fermentation
Tartaric acid biosynthesis
Protein metabolism and modification
Protein folding
Chaperone-mediated protein folding
HSP-mediated protein folding
Protein folding.Disulfide bond rearrangement
Peptidyl prolyl isomerase-mediated protein folding
Protein modification
Protein deacetylation
Histone deacetylation
Protein SUMOylation
Proteolysis
Peptidase-mediated proteolysis
Metallopeptidase-mediated proteolysis
Subtilase-mediated proteolysis
Vacuolar proteolysis
Protease inhibition
Translation
Amino acid activation
Translational elongation
Translational termination

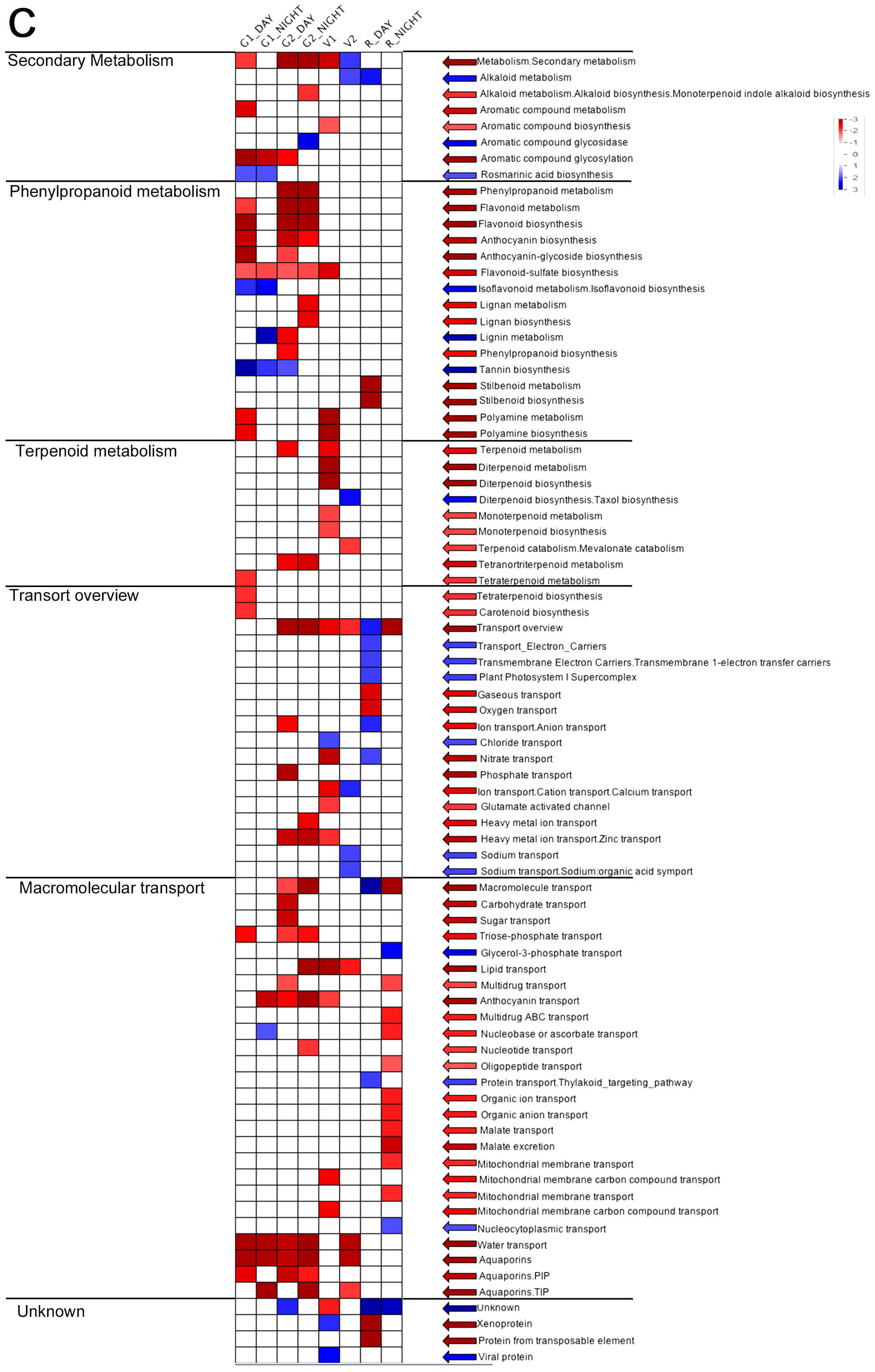
C
G1_DAY
G1_NIGHT
G2_DAY
G2_NIGHT
V1
V2
R_DAY
R_NIGHT
Secondary Metabolism
Phenylpropanoid metabolism
Terpenoid metabolism
Transort overview
Macromolecular transport
Unknown
Metabolism.Secondary metabolism
Alkaloid metabolism
Alkaloid metabolism.Alkaloid biosynthesis.Monoterpenoid indole alkaloid biosynthesis
Aromatic compound metabolism
Aromatic compound biosynthesis
Aromatic compound glycosidase
Aromatic compound glycosylation
Rosmarinic acid biosynthesis
Phenylpropanoid metabolism
Flavonoid metabolism
Flavonoid biosynthesis
Anthocyanin biosynthesis
Anthocyanin-glycoside biosynthesis
Flavonoid-sulfate biosynthesis
Isoflavonoid metabolism.Isoflavonoid biosynthesis
Lignan metabolism
Lignan biosynthesis
Lignin metabolism
Phenylpropanoid biosynthesis
Tannin biosynthesis
Stilbenoid metabolism
Stilbenoid biosynthesis
Polyamine metabolism
Polyamine biosynthesis
Terpenoid metabolism
Diterpenoid metabolism
Diterpenoid biosynthesis
Diterpenoid biosynthesis.Taxol biosynthesis
Monoterpenoid metabolism
Monoterpenoid biosynthesis
Terpenoid catabolism.Mevalonate catabolism
Tetranortriterpenoid metabolism
Tetraterpenoid metabolism
Tetraterpenoid biosynthesis
Carotenoid biosynthesis
Transport overview
Transport_Electron_Carriers
Transmembrane Electron Carriers.Transmembrane 1-electron transfer carriers
Plant Photosystem I Supercomplex
Gaseous transport
Oxygen transport
Ion transport.Anion transport
Chloride transport
Nitrate transport
Phosphate transport
Ion transport.Cation transport.Calcium transport
Glutamate activated channel
Heavy metal ion transport
Heavy metal ion transport.Zinc transport
Sodium transport
Sodium transport.Sodium:organic acid symport
Macromolecule transport
Carbohydrate transport
Sugar transport
Triose-phosphate transport
Glycerol-3-phosphate transport
Lipid transport
Multidrug transport
Anthocyanin transport
Multidrug ABC transport
Nucleobase or ascorbate transport
Nucleotide transport
Oligopeptide transport
Protein transport.Thylakoid_targeting_pathway
Organic ion transport
Organic anion transport
Malate transport
Malate excretion
Mitochondrial membrane transport
Mitochondrial membrane carbon compound transport
Mitochondrial membrane transport
Mitochondrial membrane carbon compound transport
Nucleocytoplasmic transport
Water transport
Aquaporins
Aquaporins.PIP
Aquaporins.TIP
Unknown
Xenoprotein
Protein from transposable element
Viral protein
-3
-2
-1
0
1
2
3
